# Identification and validation of candidate clinical signatures of apolipoprotein L isoforms in hepatocellular carcinoma

**DOI:** 10.1038/s41598-023-48366-0

**Published:** 2023-11-28

**Authors:** Xiang-Kun Wang, Yu-Xiang Guo, Miao Wang, Xu-Dong Zhang, Zhong-Yuan Liu, Mao-Sen Wang, Kai Luo, Shuai Huang, Ren-Feng Li

**Affiliations:** 1 Departments of Hepatobiliary and Pancreatic Surgery, The First Affiliated Hospital of Zhengzhou University, Zhengzhou, 450052 Henan Province People’s Republic of China; 2 Department of Gastrointestinal Oncology, Nanyang Second General Hospital, Nanyang, 473009 Henan Province People’s Republic of China

**Keywords:** Cancer models, Cancer screening, Tumour biomarkers, Liver cancer, Gene expression

## Abstract

Hepatocellular carcinoma (HCC) is a lethal malignancy worldwide with an increasing number of new cases each year. Apolipoprotein (APOL) isoforms have been explored for their associations with HCC.The GSE14520 cohort was used for training data; The Cancer Genome Atlas (TCGA) database was used for validated data. Diagnostic, prognostic significance and mechanisms were explored using these cohorts. Risk score models and nomograms were constructed using prognosis-related isoforms and clinical factors for survival prediction. Oncomine and HCCDB databases were further used for validation of diagnostic, prognostic significance. APOL1, 3, and 6 were differentially expressed in two cohorts (all *P* ≤ 0.05). APOL1 and APOL6 had diagnostic capacity whereas APOL3 and APOL6 had prognostic capacity in two cohorts (areas under curves [AUCs] > 0.7, *P* ≤ 0.05). Mechanism studies demonstrated that APOL3 and APOL6 might be involved in humoral chemokine signaling pathways (all *P* ≤ 0.05). Risk score models and nomograms were constructed and validated for survival prediction of HCC. Moreover, diagnostic values of APOL1 and weak APOL6 were validated in Oncomine database (AUC > 0.700, 0.694); prognostic values of APOL3 and APOL6 were validated in HCCDB database (all *P* < 0.05). Differentially expressed APOL1 and APOL6 might be diagnostic biomarkers; APOL3 and APOL6 might be prognostic biomarkers of RFS and OS for HCC via chemokine signaling pathways.

## Introduction

Global cancer statistics in 2018 indicated that liver cancer in both sexes ranked seventh in morbidity as approximately 4.7% of 36 cancer types and 841,080 new diagnosis in 185 countries. Liver cancer is third in mortality of approximately 8.2% of 36 cancer types with 781,631 cancer deaths in 185 countries^1^. Higher incidence and mortality by roughly 2- to 3-times are seen in males compared to females in most world regions^1^. Accounting for most types of primary liver cancer were hepatocellular carcinoma (HCC) at 75–85% and intrahepatic cholangiocarcinoma at 10–15%^1^. Factors such as chronic hepatitis B virus (HBV) or hepatitis C virus infection, food contamination with aflatoxin, high alcohol consumption, cirrhosis, male sex, smoking and HCC family history are risk elements for HCC tumorigenesis and progression^2, 3^. In addition to external risk factors, genetic gene alterations are recognized etiological constituents of HCC initiation and progression^4^. Although advances have occurred in surgical resection, which is the best approach for HCC treatment, the prognosis of HCC remains poor with an approximately 30% 5-year survival rate^5^. Furthermore, more than 70% of developed tumor recurrences occur at 5 years^6, 7^. Some potential biomarkers have been identified for HCC diagnosis and prognosis^8, 9^. However, these biomarkers need further validation in varied populations. Therefore, identification of new biomarkers for HCC early diagnosis and prognosis is crucial for better patient survival.

The apolipoprotein (APO) superfamily contains 10 subfamilies: APOA, APOB, APOC, APOD, APOE, APOF, APOH, APOL, APOM, and APOO (https://www.genenames.org/data/genegroup/#!/group/405). The APOL subfamily has six isoforms: APOL1, APOL2, APOL3, APOL4, APOL5, and APOL6^10^. APOs are critical for the development of high-density lipoprotein (HDL) and low-density lipoprotein complexes^11^. APOL1 binds to HDL^12^. As a major source of HDL production and circulating APOs, the liver is pivotal for the circulating pool of APOL1^13, 14^. In addition, APOL1 induces autophagy-mediated cell death independent of caspase-mediated apoptosis and can be a general autophagy mediator^15^. APOL2 has an anti-apoptotic function in interferon-γ-induced cytotoxicity in human bronchial epithelial cells^16^. The APOL3 region on chromosome 22q12 was a risk locus in a family-based association analysis of 42 families with hereditary prostate cancer^17^. Single nucleotide polymorphisms and haplotypes in APOL1, 2 and 4, located on chromosome 22q12.3–13.1, are associated with schizophrenia in African-American, European-American, Chinese and Japanese populations^18^. APOL5 is reported rare with disease. Overexpression of wild-type APOL6 leads to mitochondrial-mediated apoptosis in DLD-1 cells, characterized by release of cytochrome c and Smac/DIABLO from mitochondria and activation of caspase 9^19^. APOL family isoforms transport HDL in cell membranes are important for the development and maintenance of membrane structure and function^20, 21^. Therefore, we hypothesized that aberrant expression of APOLs may be associated with HCC tumorigenesis and progression because of APOL isoforms involvement in membrane structure and function and as a major source of HDL production and circulation of APOs in the liver. We conducted this study to explore the potential roles of APOL isoforms in HCC.

## Material and methods

### Patient data and ethical approval

Gene profile data of GSE14520 was used for a training cohort. To avoid batch effects, only the platform of GPL3921 in this dataset, including 212 HBV-HCC patients, was used for analysis (https://www.ncbi.nlm.nih.gov/geo/query/acc.cgi?acc=GSE14520)^22, 23^. A total of 370 HCC patients from The Cancer Genome Atlas (TCGA) database were used for validation (https://cancergenome.nih.gov/). GSE14520 dataset had its features with HBV-related, most cirrhosis background, and Asian race, while TCGA dataset had its features with most patients over 40 years old, Asian race with less than 45%, roughly 50% patients with BMI ≥ 24.

### Analysis of diagnostic and prognostic significance

Expression of APOL1-6 mRNA was used for diagnostic and prognostic analysis. Expression of APOL1-6 mRNA in HCC and nontumor tissues was used for diagnostic capacity assessment in the GSE14520 and TCGA cohorts. Expression of APOL1-6 in HCC tissues was used for prognosis assessment in the GSE14520 and TCGA cohorts. Expression of APOL1-6 was divided into low and high by median expression levels. Prognosis-related genes were combined for joint-effect analysis for overall survival (OS) and recurrence-free survival (RFS).

### Mechanism exploration of prognosis-related genes in genome-wide

Identified prognosis-related genes were explored for potential mechanisms in HCC with genome-wide gene set enrichment analysis (GSEA). GSEA was performed using gene ontologies (GO) of biological processes (BP, c5.bp.b6.1.symbols.gmt), cellular component (CC, c5.cc.v6.1.symbols.gmt), molecular function (MF, c5.mf.v6.1.symbols.gmt), and Kyoto Encyclopedia of Genes and Genomes pathways (KEGG, c2.cp.kegg.v6.1.symbols.gmt). *P* values ≤ 0.05 and false discovery rate ≤ 0.25 were considered significant.

### Risk score model and nomogram construction

Risk score models were used for HCC prognosis predictions by gene expression. A risk score model was constructed using coefficients and expressions of different genes using the formula: risk scores = expression of gene_1_ x β_1_ (coefficient) + expression of gene_2_ x β_2_ (coefficient) + … + expression of gene_n_ x β_n_ (coefficient)^24–26^. Risk score models included risk score rankings, survival status, expression heatmaps, Kaplan–Meier plots and time-dependent receiver operating characteristic curves.

Nomograms were constructed for HCC prediction of clinical factors and gene expression. Prognosis-related genes and prognosis-related clinical factors were used in nomograms. Different expression levels and factors indicated different points and total points equal to the sum of all points.

### Co-expression matrixes and interaction networks

Co-expression matrixes of APOL1-6 were constructed using mRNA expression in the GSE14520 and TCGA cohorts. Co-expression networks of gene–gene interaction (GGI) of APOL1-6 were constructed using the geneMANIA plugin of Cytoscape software^27, 28^. Chemical association networks were constructed using APOL1-6 and visualized for APOL1-6 and chemicals using the STITCH website (http://stitch.embl.de/)^29^. Visualized GO term interaction networks were constructed using the BinGO plugin of Cytoscape software^30^.

### Validation of diagnostic analysis and prognosis significance by Oncomine and HCCDB databases

Differential expressions and diagnostic significance of APOL isoforms were further validated using Wurmbach dataset in Oncomine database (https://www.oncomine.org/resource/main.html)^31^. Furthermore, prognosis-related APOL isoforms, including OS and RFS, in TCGA and GSE14520 datasets were further validated in HCCDB database (http://lifeome.net/database/hccdb/home.html)^32^.

### Statistical analysis

Survival analysis was by SPSS software version 24 (IBM, Chicago, IL). Scatter plots and the Kaplan–Meier method were generated using GraphPad 7.0. Calculations of 95% confidence intervals and hazard ratios were by univariate and multivariate Cox regression models. Median survival time and log-rank *P* value were calculated by the Kaplan–Meier method. RT-PCR was validated using paired *t*-tests. A *P* value ≤ 0.05 was considered significant.

## Results

### Demographic characteristics and mRNA expression analysis

The GSE14520 cohort contained 212 patients with HBV-related HCC. The TCGA cohort contained 370 patients with HCC. Characteristics of the cohorts are in our previous report^33^.

**Figure 1:**
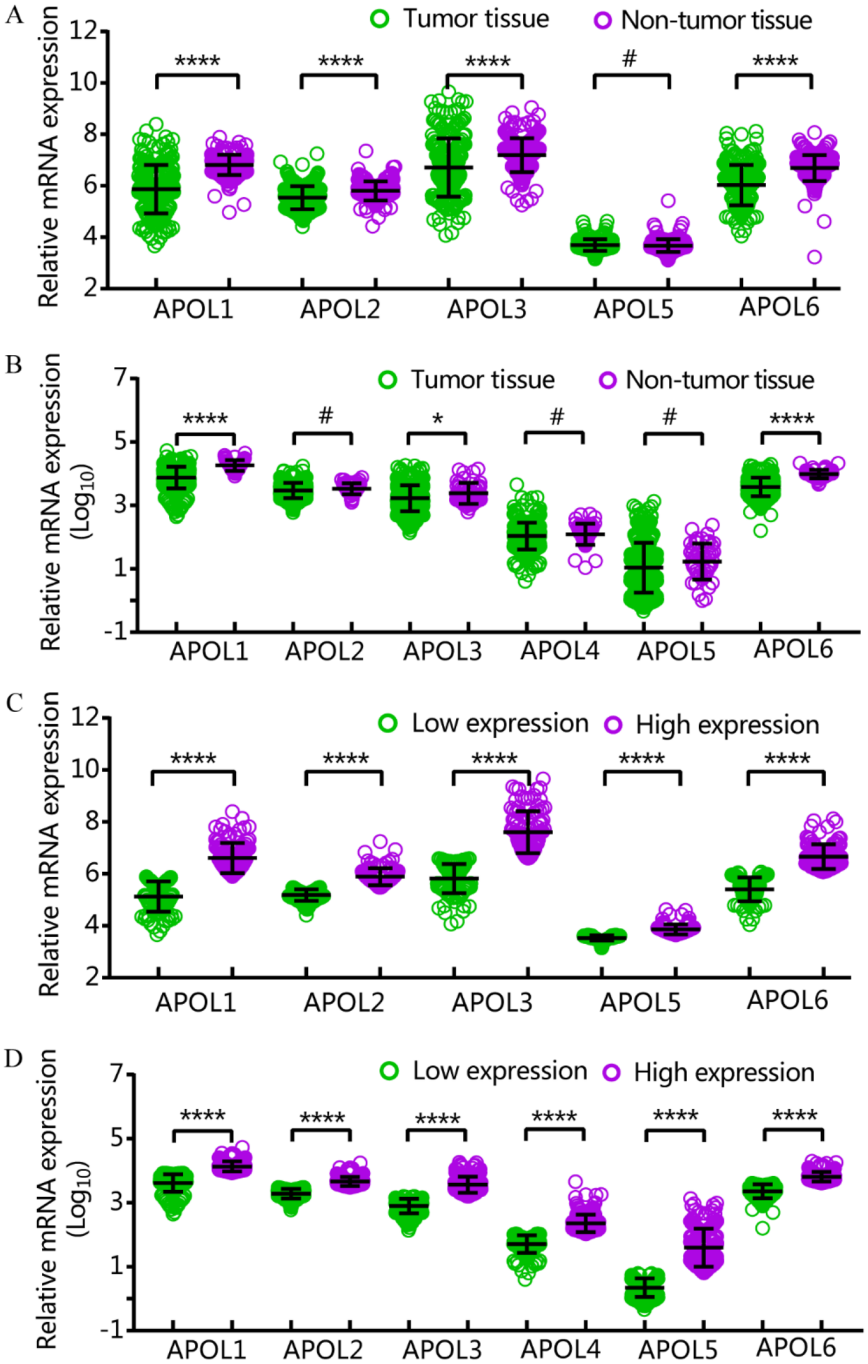
APOL1-6 mRNA in TCGA and GSE14520 cohorts. (**A**–**B**): APOL1-6 mRNA of tumor and nontumor tissues in GSE14520 and TCGA cohorts. (**C**–**D**): APOL1-6 mRNA in low and high expression groups in GSE14520 and TCGA cohorts.

APOL1, 2, 3, and 6 were differentially expressed in tumor and non-tumor tissues in the GSE14520 cohort. APOL1, 3 and 6 were differentially expressed in tumor and non-tumor tissues in the TCGA cohort (Fig. 1A,B). All APOL isoforms were differentially expressed in low and high expression groups in both cohorts (Fig. 1C,D). APOL4 was not included in the GSE14520 cohort.

### Diagnostic capacity and prognostic significance analysis

**Figure 2:**
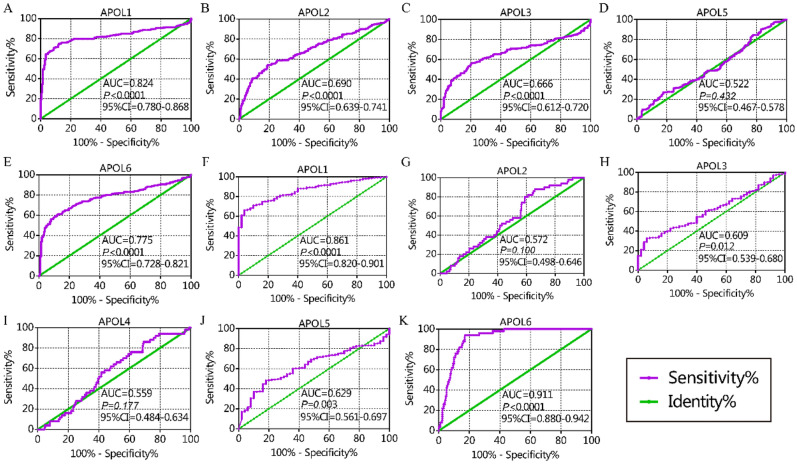
Diagnostic capacity of APOL1-6 in GSE14520 and TCGA cohorts. (**A**–**E**): Diagnostic capacity of APOL1, 2, 3, 5, and 6 in the GSE14520 cohort. (**F**–**K**): Diagnostic capacity of APOL1-6 in the TCGA cohort.

From the diagnostic capacity analysis, in the GSE14520 cohort, APOL1 and APOL6 had diagnostic significance for HCC (APOL1: area under curve [AUC] 0.824, *P* < 0.0001; APOL6: AUC 0.775, *P* < 0.0001, Fig. 2A,E). In the TCGA cohort, APOL1 and APOL6 had diagnostic significance for HCC (APOL1: AUC 0.824, *P* < 0.0001; APOL6: AUC 0.911, *P* < 0.0001, Fig. 2F,K). Others showed no or weak diagnostic capacity for HCC (Fig. 2).

**Figure 3:**
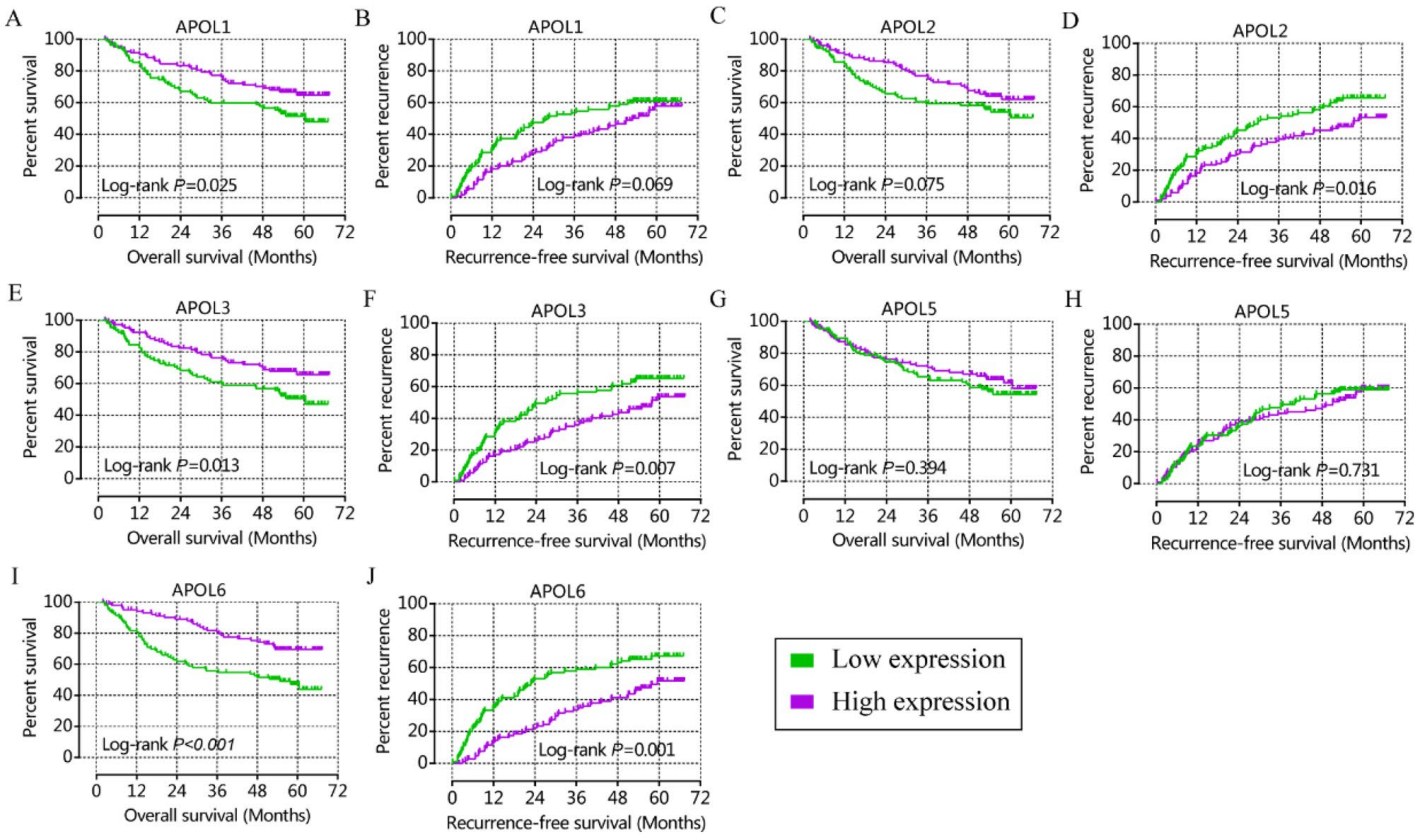
Kaplan–Meier plots of overall survival and recurrence-free survival for APOL1, 2, 3, 5 and 6 in the GSE14520 cohort. (**A**, **C**, **E**, **G**, **I**): Kaplan–Meier plots of overall survival for APOL1, 2, 3, 5, and 6; (**B**, **D**, **F**, **H**, **J**): Kaplan–Meier plot of recurrence-free survival for APOL1-6.

**Figure 4:**
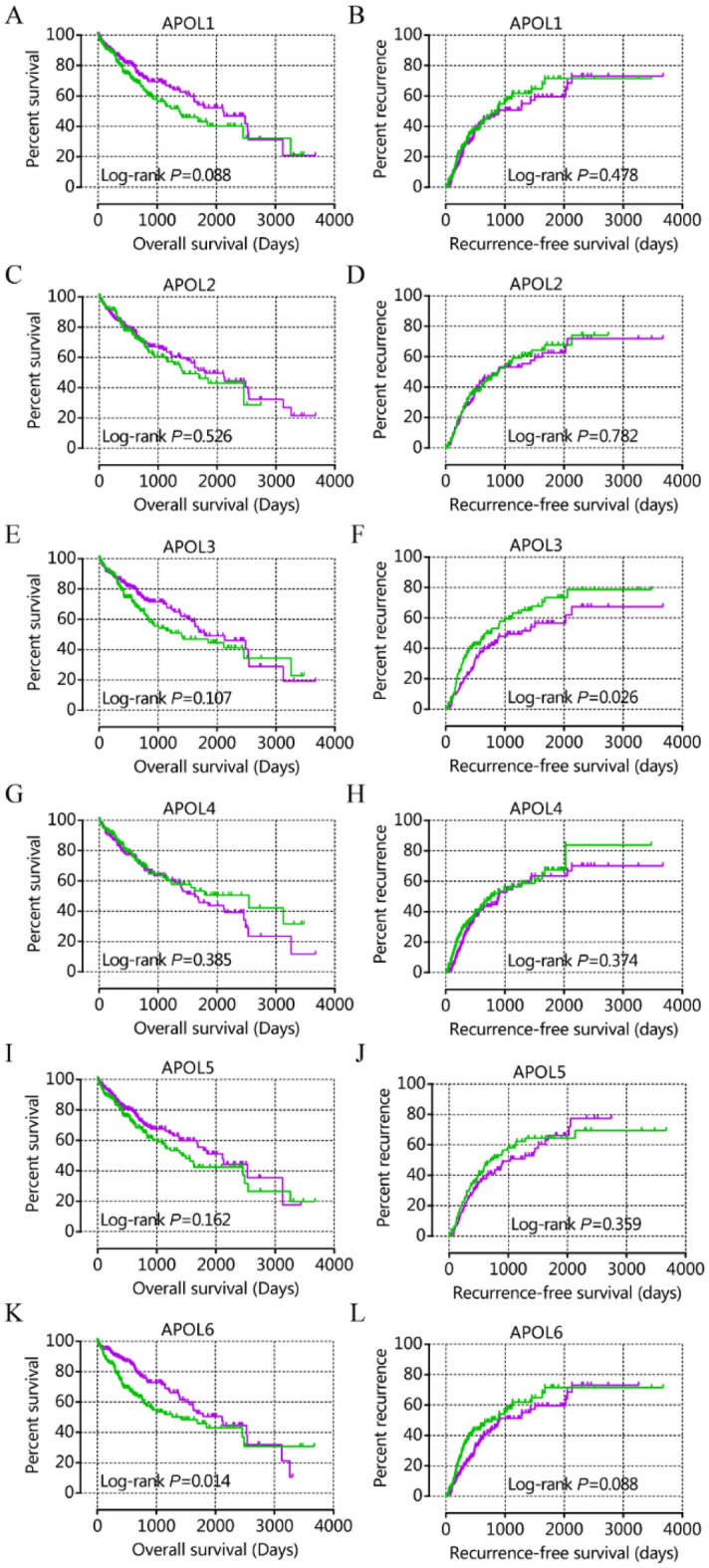
Kaplan–Meier plots of overall survival and recurrence-free survival for APOL1-6 in the TCGA cohort. (**A**, **C**, **E**, **G**, **I**, **K**): Kaplan–Meier plots for overall survival for APOL1-6; (**B**, **D**, **F**, **H**, **J**, **L**): Kaplan–Meier plots for recurrence-free survival for APOL1-6.

From prognostic significance analysis, in the GSE14520 cohort, APOL1, APOL3 and APOL6 showed prognostic value by univariate analysis. APOL3 and APOL6 showed prognostic value for OS by multivariate analysis (Table 1, Fig. 3). APOL2, APOL3 and APOL6 showed prognostic value for RFS in both univariate and multivariate analysis (Table 1, Fig. 3). In the TCGA cohort, only APOL6 showed prognostic value for OS in univariate and multivariate analysis (Table 2, Fig. 4). APOL3 and APOL4 had prognostic value for RFS in multivariate analysis (Table 2).

**Table 1:** Prognostic analysis of APOL isoforms for patient survival in GSE14520 cohort.

Type	Variables	Patients	Patients survival					
		(n = 212)	No. of event	MST (month)	HR (95%CI)	Crude *P* value	HR (95%CI)	Adjusted *P* value^ɸ^
OS	APOL1					**0.025**		0.125
	Low expression	106	48	60.5	Reference		Reference	
	High expression	106	34	NA	**0.605 (0.390–0.940)**		0.700 (0.444–1.104)	
	APOL2					0.075		0.164
	Low expression	106	46	NA	Reference		Reference	
	High expression	106	36	NA	0.672 (0.434–1.040)		0.729 (0.467–1.137)	
	APOL3					**0.013**		**0.032**
	Low expression	106	49	60.5	Reference		Reference	
	High expression	106	33	NA	**0.571 (0.367–0.888)**		**0.610 (0.387–0.959)**	
	APOL5					0.394		0.242
	Low expression	106	44	NA	Reference		Reference	
	High expression	106	38	NA	0.828 (0.536–1.278)		0.764 (0.487–1.199)	
	APOL6					** < 0.001**		**0.007**
	Low expression	106	53	54.8	Reference		Reference	
	High expression	106	29	NA	**0.434 (0.276–0.684)**		**0.527 (0.331–0.840)**	
RFS	APOL1					0.069		0.145
	Low expression	106	62	28.2	Reference		Reference	
	High expression	106	54	53.0	0.713 (0.495–1.027)		0.757 (0.520–1.101)	
	APOL2					**0.016**		**0.023**
	Low expression	106	66	29.9	Reference		Reference	
	High expression	106	50	57.9	**0.635 (0.439–0.917)**		**0.648 (0.446–0.942)**	
	APOL3					**0.007**		**0.018**
	Low expression	106	66	26.9	Reference		Reference	
	High expression	106	50	57.9	**0.601 (0.416–0.869)**		**0.634 (0.435–0.924)**	
	APOL5					0.731		0.641
	Low expression	106	59	40.1	Reference		Reference	
	High expression	106	57	51.1	0.938 (0.652–1.351)		0.916 (0.633–1.326)	
	APOL6					**0.001**		**0.004**
	Low expression	106	68	23.0	Reference		Reference	
	High expression	106	48	59.5	**0.519 (0.358–0.751)**		**0.571 (0.392–0.833)**	

ɸ: *P* values were adjusted for tumor size, cirrhosis, AFP and BCLC stage in OS and were adjusted for gender, cirrhosis and BCLC stage in RFS; Bold indicates significant *P* values.

NA: not available; MST: median survival time; HR: hazard ratio; 95%CI: 95% confidence interval; OS: overall survival; RFS: recurrence-free survival.

**Table 2:** Prognostic analysis of APOL isoforms for patient survival in TCGA cohort.

Type	Variables	Patients	Overall survival					
		(n = 370)	No. of event	MST (days)	HR (95%CI)	Crude *P* value	HR (95%CI)	Adjusted *P* value^Ѱ^
OS	APOL1					0.088		0.123
	Low expression	185	68	1372	Reference		Reference	
	High expression	185	62	2116	0.740 (0.523–1.046)		0.735 (0.498–1.087)	
	APOL2					0.526		0.709
	Low expression	185	62	1423	Reference		Reference	
	High expression	185	68	1791	0.893 (0.628–1.268)		0.927 (0.624–1.378)	
	APOL3					0.107		0.126
	Low expression	185	68	1397	Reference		Reference	
	High expression	185	62	1791	0.752 (0.533–1.063)		0.737 (0.499–1.090)	
	APOL4					0.385		0.961
	Low expression	185	59	2542	Reference		Reference	
	High expression	185	71	1624	1.166 (0.825–1.648)		1.010 (0.682–1.494)	
	APOL5					0.162		0.974
	Low expression	185	71	1490	Reference		Reference	
	High expression	185	59	2116	0.781 (0.552–1.104)		0.994 (0.675–1.462)	
	APOL6					**0.014**		**0.004**
	Low expression	185	72	1423	Reference		Reference	
	High expression	185	58	2116	**0.646 (0.457–0.914)**		**0.565 (0.383–0.835)**	
RFS	APOL1					0.478		0.224
	Low expression	159	71	875	Reference		Reference	
	High expression	159	68	903	0.886 (0.635–1.237)		0.771 (0.507–1.172)	
	APOL2					0.782		0.836
	Low expression	164	73	893	Reference		Reference	
	High expression	154	66	879	0.954 (0.683–1.332)		0.956 (0.624–1.465)	
	APOL3					**0.026**		**0.026**
	Low expression	159	77	776	Reference		Reference	
	High expression	159	62	1286	**0.684 (0.489–0.956)**		**0.619 (0.405–0.945)**	
	APOL4					0.374		
	Low expression	162	73	776	Reference		Reference	**0.050**
	High expression	156	66	903	0.859 (0.614–1.201)		**0.647 (0.409–1.001)**	
	APOL5					0.359		0.605
	Low expression	156	71	701	Reference		Reference	
	High expression	162	68	1032	0.856 (0.613–1.194)		0.895 (0.588–1.362)	
	APOL6					0.088		0.118
	Low expression	158	71	776	Reference		Reference	
	High expression	160	68	912	0.748 (0.536–1.044)		0.715 (0.470–1.089)	

Ѱ: *P* values were adjusted for HBV infection, tumor stage, and radical resection in OS and were adjusted for HBV infection, tumor stage, radical resection and vascular invasion in RFS; Bold indicates significant P values.

NA: not available; MST: median survival time; HR: hazard ratio; 95%CI: 95% confidence interval; OS: overall survival; RFS: recurrence-free survival.

### Analysis of combined prognosis-related genes

Prognosis-related genes were used for combined analysis. APOL2, APOL3, and APOL6 were combined for OS and APOL3 and APOL6 were combined for RFS in the GSE14520 cohort (Table 3, Figure S1A-E). APOL3 and APOL4 were combined for RFS in TCGA cohort (Table S1, Figure S1F). Groups containing two poor prognosis indicators had the worst survival times whereas groups with two good prognosis indicators had the best survival times.

**Table 3:** Joint-effect analysis of *APOL2*, *APOL3* and *APOL6* for prognosis analysis in GSE14520 cohort.

Group	*APOL2*	*APOL3*	*APOL6*	Prognosis	MST (Months)	Adjusted HR (95%CI)	Adjusted *P* value^&^
				Events/total			
RFS							
I	Low	Low		47/71	23.0	Reference	**0.018**
II	Low	High		38/70	51.1	0.731 (0.469–1.139)	0.166
	High	Low					
III	High	High		31/71	59.5	**0.509 (0.320–0.812)**	**0.005**
1	Low		Low	49/72	19.6	Reference	**0.004**
2	Low		High	36/68	51.6	**0.607 (0.393–0.938)**	**0.025**
	High		Low				
3	High		High	31/72	NA	**0.474 (0.299–0.751)**	**0.002**
A		Low	Low	50/77	22.8	Reference	**0.007**
B		Low	High	34/58	32.6	0.724 (0.464–1.130)	0.155
		High	Low				
C		High	High	32/77	NA	**0.485 (0.308–0.763)**	**0.002**
a	Low	Low	Low	41/57	19.4	Reference	**0.001**
b	Low	High	Low	23/49	NA	**0.479 (0.285–0.807)**	**0.006**
	Low	Low	High				
	High	Low	Low				
c	High	High	Low	31/49	36.6	**0.676 (0.422–1.084)**	**0.104**
	High	Low	High				
	Low	High	High				
d	High	High	High	21/57	NA	**0.343 (0.200–0.591)**	** < 0.001**
OS							
●		Low	Low	39/77	51.6	Reference	**0.015**
●●		Low	High	24/58	NA	0.701 (0.418–1.176)	0.179
		High	Low				
●●●		High	High	19/77	NA	**0.437 (0.248–0.770)**	**0.004**

^&^: *P* values were adjusted for gender, cirrhosis and BCLC stage; Bold indicates significant *P* values.

RFS: recurrence-free survival; OS: overall survival; NA: not available; MST: median survival time; HR: hazard ratio; 95%CI: 95% confidence interval; OS: overall survival; RFS: recurrence-free survival.

### Prospective molecular mechanism exploration by GSEA

**Figure 5:**
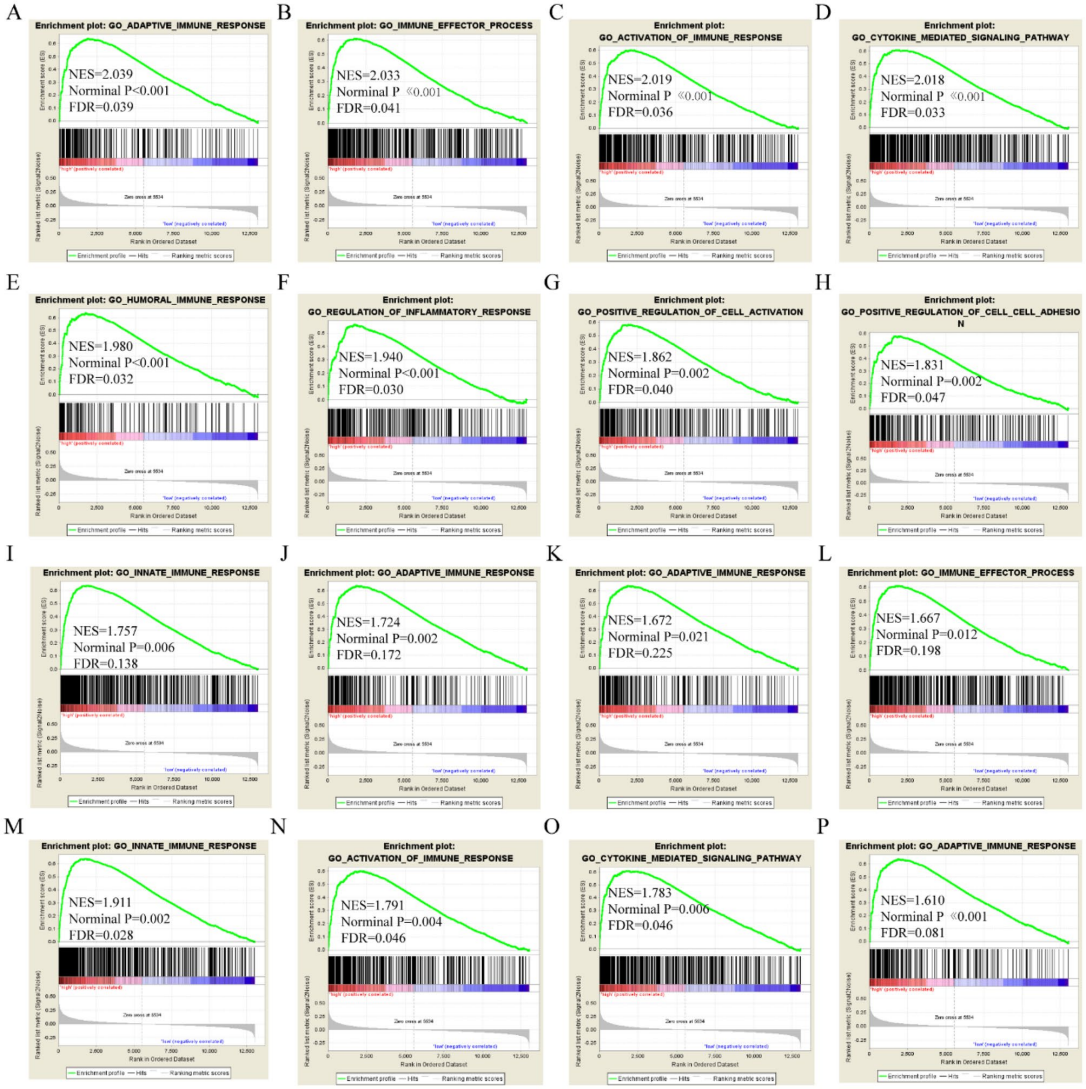
Gene ontology and KEGG pathway results for APOL3 in the GSE14520 cohort. (**A**–**L**): Gene ontology results for the APOL3 gene; (**M**–**P**): KEGG pathway results for the APOL3 gene.

GSEA was performed to explore prospective molecular genome-wide mechanisms of APOL isoform involvement in HCC. APOL3 was involved in the adaptive immune response, immune effector processes, humoral immune response, positive cell activation, regulation of inflammatory responses, and cytokine-mediated signaling pathways by GO terms in the GSE14520 cohort (Fig. 5A–L). APOL3 was found to be involved in cell adhesion molecular cams, chemokine-signaling pathways, type 1 diabetes mellitus, and fatty acid metabolism by KEGG pathway in the GSE14520 cohort (Fig. 5M–P). APOL6 was found to be involved in the humoral immune response, fatty acid metabolism, immune effector processes, cytokine-mediated signaling pathways, and drug metabolism cytochrome P450 in the GSE14520 cohort (Figure S2).

**Figure 6:**
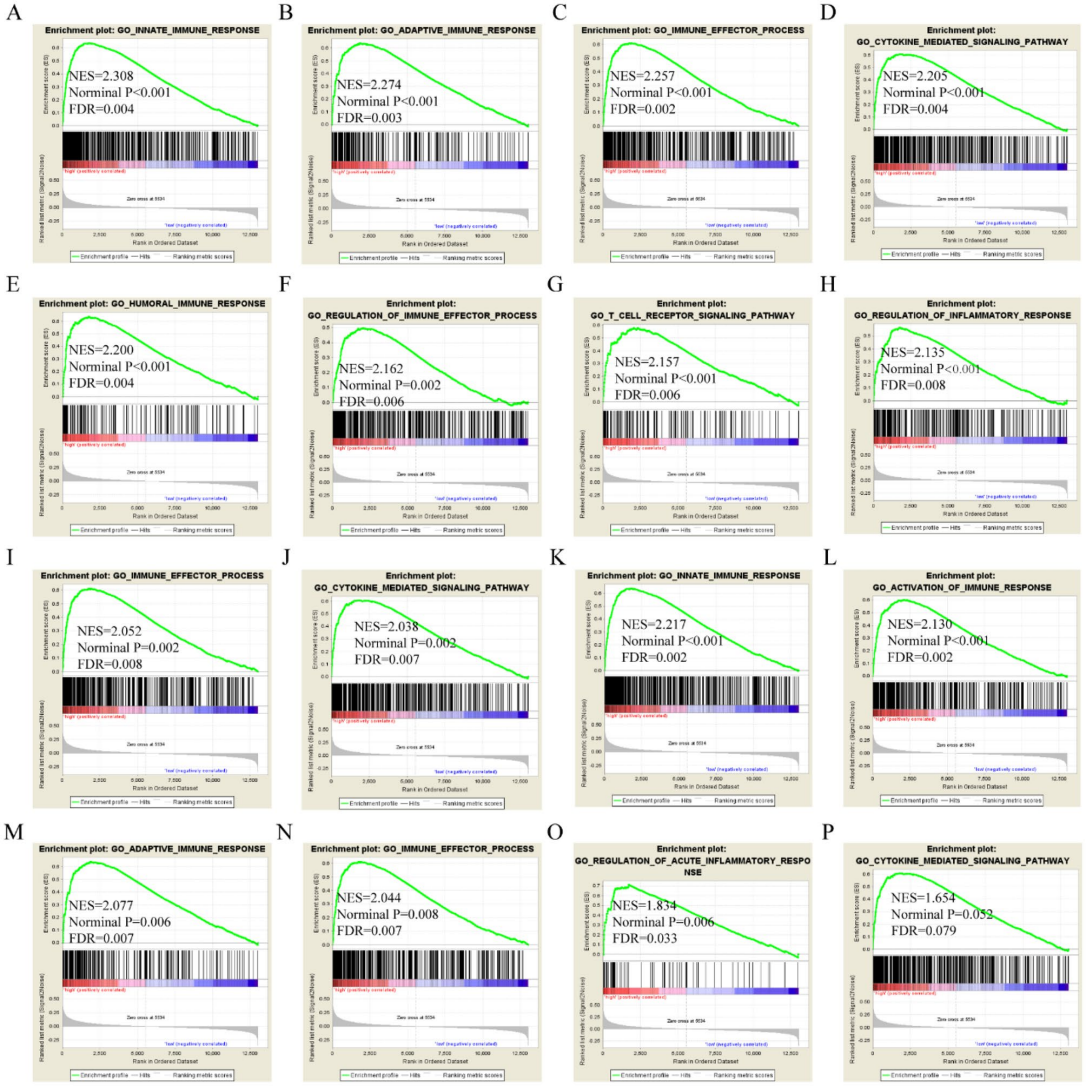
Gene ontology and KEGG pathway results for APOL3 in the TCGA cohort. (**A**–**L**): Gene ontology results for the APOL3 gene. (**M**–**P**): KEGG pathway results for the APOL3 gene.

APOL3 was found to be involved in B-cell mediated immunity, activation of the immune response, the adaptive immune response, the humoral immune response, cytokine-mediated signaling pathways, chromosome centromeric region, histone binding, and chromatin binding by GO terms in the TCGA cohort (Fig. 6A–L). APOL3 was found to be involved in cell adhesion molecular cams, type 1 diabetes mellitus, chemokine signaling pathways, and fatty acid metabolism by KEGG pathways in the TCGA cohort (Fig. 6M–P). APOL6 was found to be involved in the immune effector response, B-cell mediated immunity, regulation of inflammatory responses, cytokine-mediated signaling pathways, JAK-STAT signaling pathways, and cell adhesion molecular cams in the TCGA cohort (Figure S3).

### Risk score model and nomogram construction

**Figure 7:**
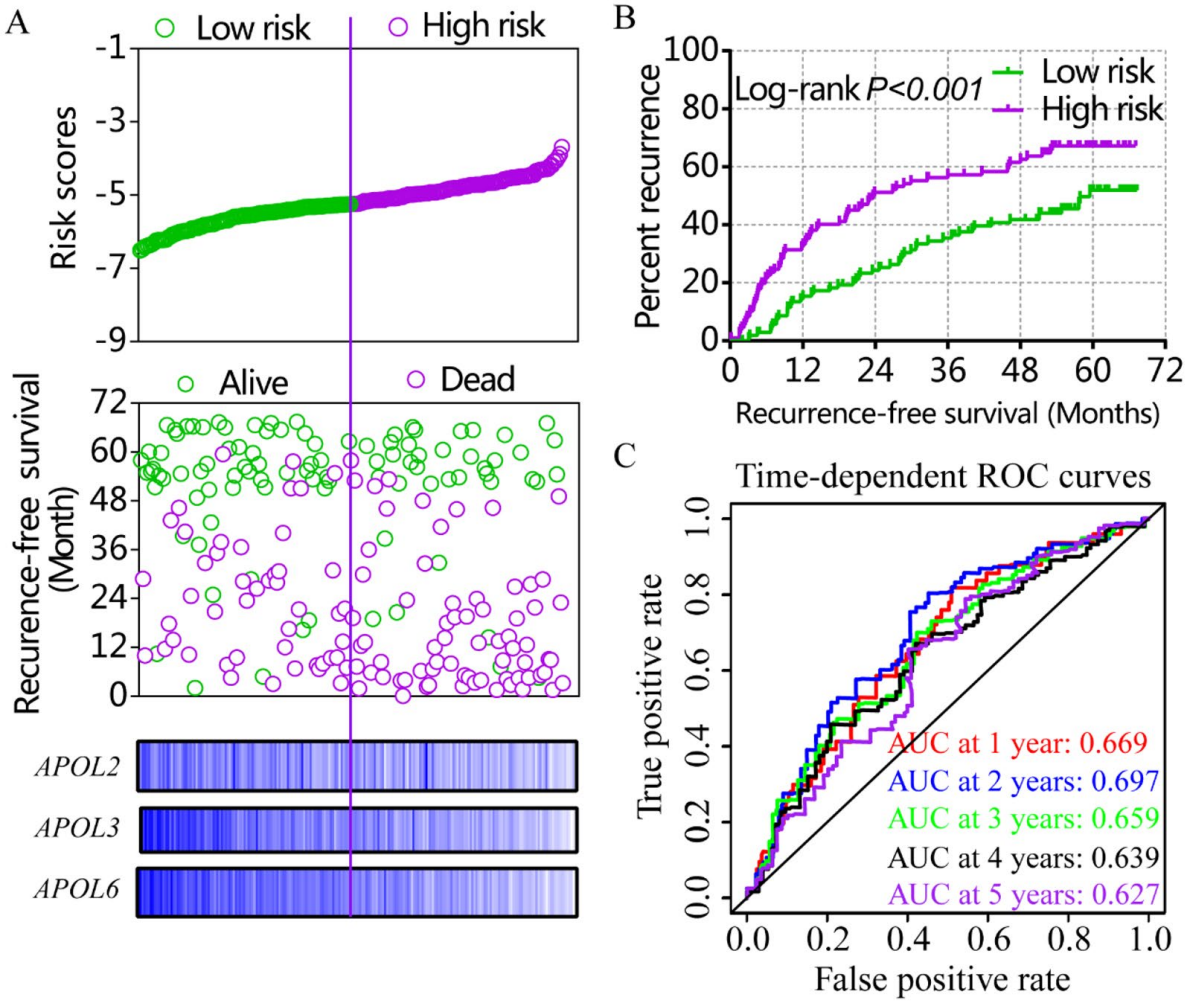
Risk score model, Kaplan–Meier plots and time-dependent receiver operative characteristic curves for recurrence-free survival in the GSE14520 cohort. (**A**): Risk score model with risk score, survival status, heatmap of APOL2, 3 and 6. (**B**): Kaplan–Meier plots by low and high recurrence-risk groups; (**C**): Time-dependent receiver operative characteristic curves for recurrence-free survival at 1, 2, 3, 4, and 5 years.

**Figure 8:**
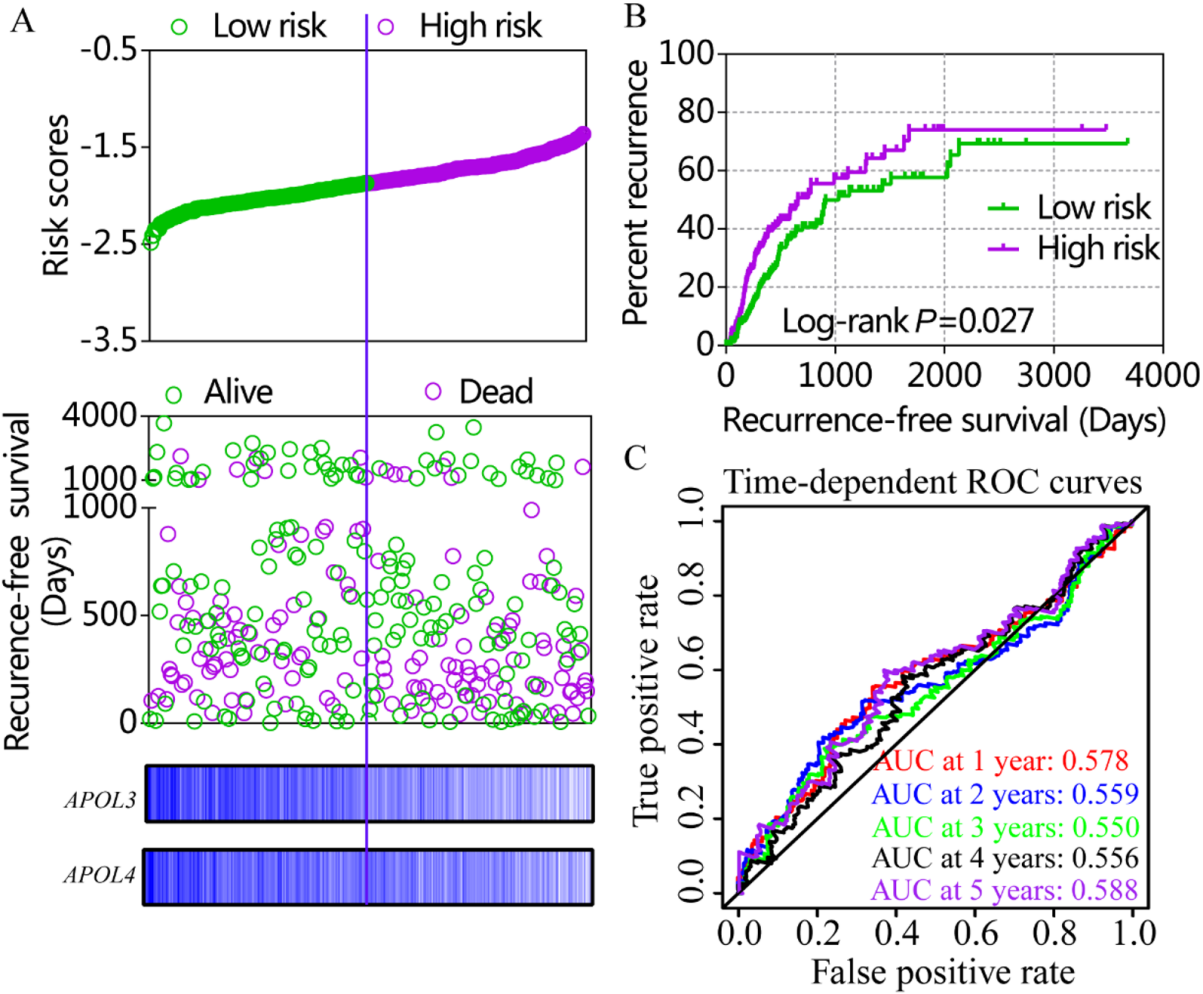
Risk score model, Kaplan–Meier plots and time-dependent receiver operative characteristic curves for recurrence-free survival in the TCGA cohort. (**A**): Risk score model with risk score, survival status, and heatmap for APOL3 and 4. (**B**): Kaplan–Meier plots by low and high recurrence-risk groups. (**C**): Time-dependent receiver operative characteristic curves for recurrence-free survival at 1, 2, 3, 4, and 5 years.

Risk score models were constructed using APOL3 and APOL6 for OS (Figure S4, Table 4) and APOL2, APOL3 and APOL6 for RFS in the GSE14520 cohort (Fig. 7, Table 4). Risk score models were constructed using APOL3 and APOL4 for RFS in the TCGA cohort (Fig. 8, Table 4). Risk score ranking, patient survival status, heat maps of APOL expression isoforms, Kaplan–Meier plots and time-dependent ROC curves for 1-, 2-, 3-, 4-, and 5-year survival were included in the models. ROC curves for the GSE14520 cohort, including OS and RFS models, showed better prognoses than for the TCGA cohort. Detailed prognostic analysis results of low- and high- risk groups were shown in Table S2.

**Table 4:** Risk score model of GSE14520 and TCGA cohort.

Dataset	Type	Variables	Coefficient	*P* values	HR	95% CI
GSE14520	OS	Tumor size	0.140	0.140	1.150	0.691–1.914
		Cirrhosis	− 1.367	− 1.367	0.255	0.062–1.048
		BCLC stage 0		< 0.001		
		Stage A	1.396	1.396	4.038	0.974–16.746
		Stage B	1.808	1.808	6.101	1.315–28.305
		Stage C	2.625	2.625	13.798	3.009–63.265
		AFP	0.209	0.209	1.232	0.788–1.926
		*APOL3*	− 0.294	− 0.294	0.745	0.458–1.212
		*APOL6*	− 0.524	− 0.524	0.592	0.358–0.980
	RFS	Gender	− 0.603	0.072	0.547	0.284–1.054
		Cirrhosis	− 0.881	0.057	0.414	0.168–1.025
		BCLC stage 0		0.001		
		Stage A	0.658	0.123	1.931	0.837–4.456
		Stage B	1.101	0.025	3.008	1.150–7.871
		Stage C	1.561	0.001	4.762	1.892–11.983
		*APOL2*	− 0.250	0.221	0.779	0.521–1.163
		*APOL3*	− 0.212	0.321	0.809	0.533–1.230
		*APOL6*	− 0.400	0.061	0.670	0.441–1.018
TCGA	RFS	Stage I		< 0.001		
		Stage II	0.986	0.002	2.682	1.440–4.993
		Stage III + IV	1.299	< 0.001	3.665	2.015–6.664
		Radical resection	1.044	0.004	2.842	1.405–5.748
		Microvascular invasion	− 0.449	0.105	0.639	0.371–1.099
		HBV infection	− 0.049	0.842	0.952	0.588–1.541
		*APOL3*	− 0.386	0.09	0.680	0.435–1.062
		*APOL4*	− 0.308	0.184	0.735	0.466–1.158

APOL2: apolipoprotein L 2; APOL3: apolipoprotein L 3; APOL4: apolipoprotein L 4; APOL6: apolipoprotein L 6; HR: hazard ratio; 95%CI: 95% confidence interval; OS: overall survival; RFS: recurrence-free survival; AFP: α-fetoprotein; BCLC: Barcelona Clinic Liver Cancer; HBV: hepatitis B virus.

**Figure 9:**
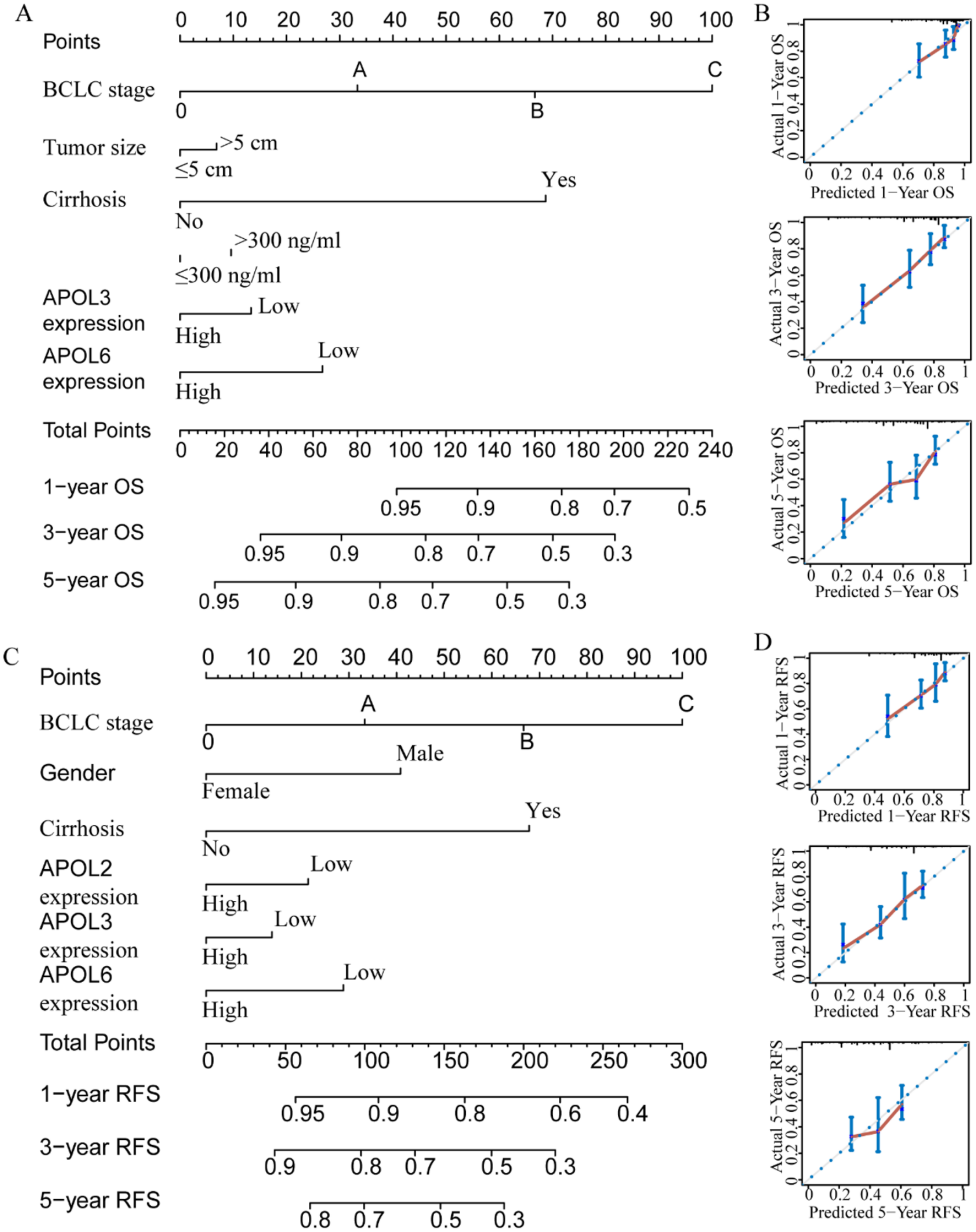
Prognosis-predicted nomograms and inner validation for 1, 3, and 5 years in the GSE14520 cohort. (**A**-–**B**): Overall survival predicting nomogram using tumor size, cirrhosis, BCLC stage, AFP levels, APOL3 and APOL6 expression for 1, 3, and 5 years and inner validation for 1, 3, and 5 years. (**C**–**D**): Recurrence-free survival predicting nomogram using sex, cirrhosis, BCLC stage APOL2, APOL3 and APOL6 expression and inner validation for 1, 3, and 5 years for 1, 3, and 5 years.

**Figure 10:**
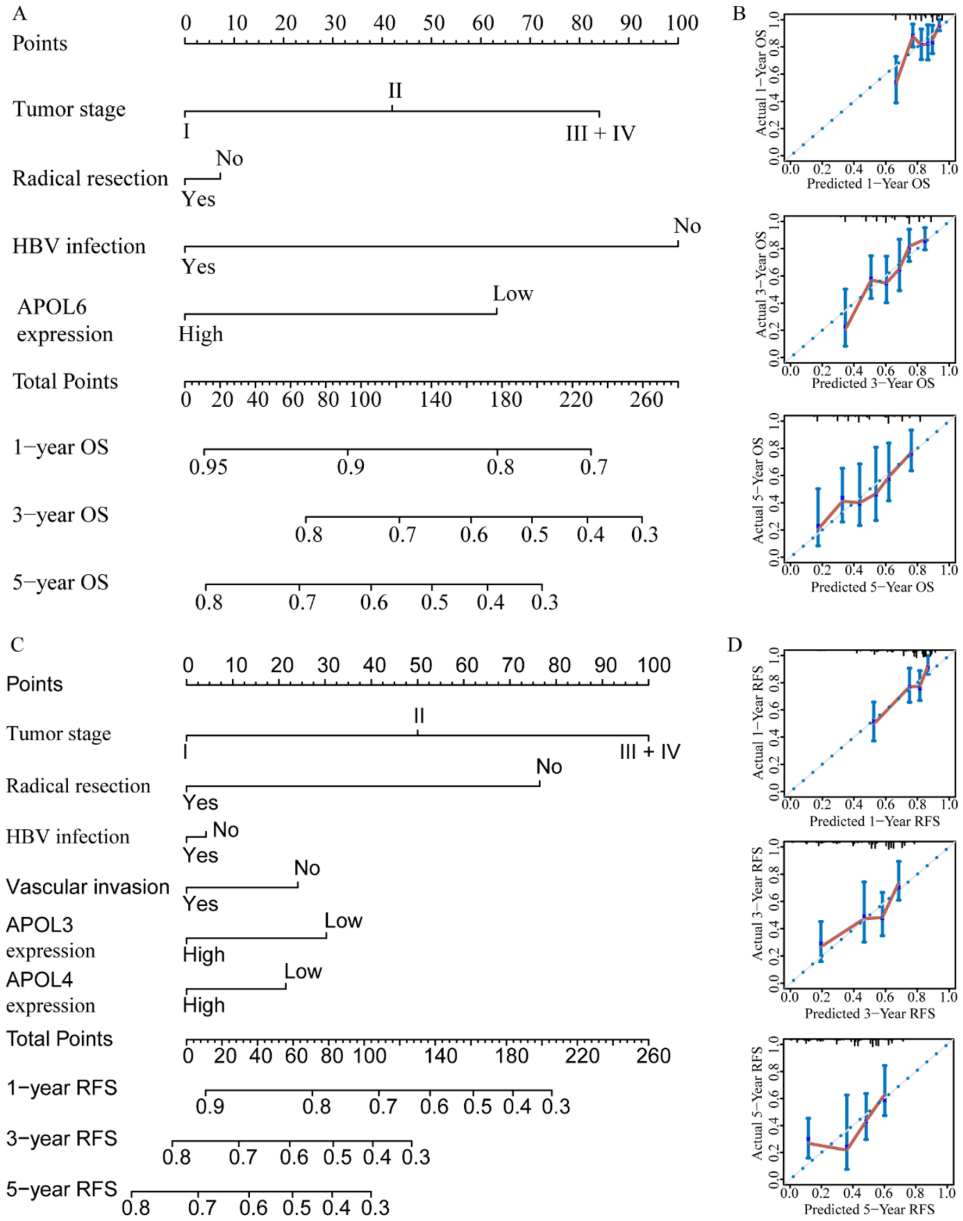
Prognosis-predicting nomograms and inner validation for 1, 3, and 5 years in the TCGA cohort. (**A**–**B**): Overall survival-predicting nomogram using tumor stage, radical resection, hepatitis B virus infection status, and APOL6 expression and inner validation for 1, 3, and 5 years. (**C**–**D**): Recurrence-free survival-predicting nomogram using tumor stage, radical resection, hepatitis B virus infection status, vascular invasion, and APOL3 and APOL4 expression and inner validation for 1, 3, and 5 years.

For the GSE14520 cohort, nomograms were constructed using tumor size, cirrhosis, α-fetoprotein (AFP), Barcelona Clinic Liver Cancer (BCLC) stage, APOL3 and APOL6 expressions for OS (Fig. 9A,B). Nomograms were constructed using sex, cirrhosis, BCLC stage, APOL2, APOL3 and APOL6 expression for RFS (Fig. 9C,D). For the TCGA cohort, nomograms were constructed using tumor stage, radical resection, HBV infection, and APOL6 expression for OS (Fig. 10A,B). Nomograms were constructed for tumor stage, radical resection, HBV infection, vascular invasion, APOL3 and APOL4 expression for RFS (Fig. 10C,D). Small tumor size; female sex; lack of cirrhosis; BCLC stage 0; high expression of APOL2, APOL3 and APOL6; and low AFP levels indicated higher survival rate in the GSE14520 cohort. Early tumor stage, radical resection, low APOL3 expression, high APOL4 and APOL6 expressions, vascular invasion and HBV infection indicated higher survival rates in the TCGA cohort.

### Co-expression, protein-chemical interaction networks and matrix

**Figure 11:**
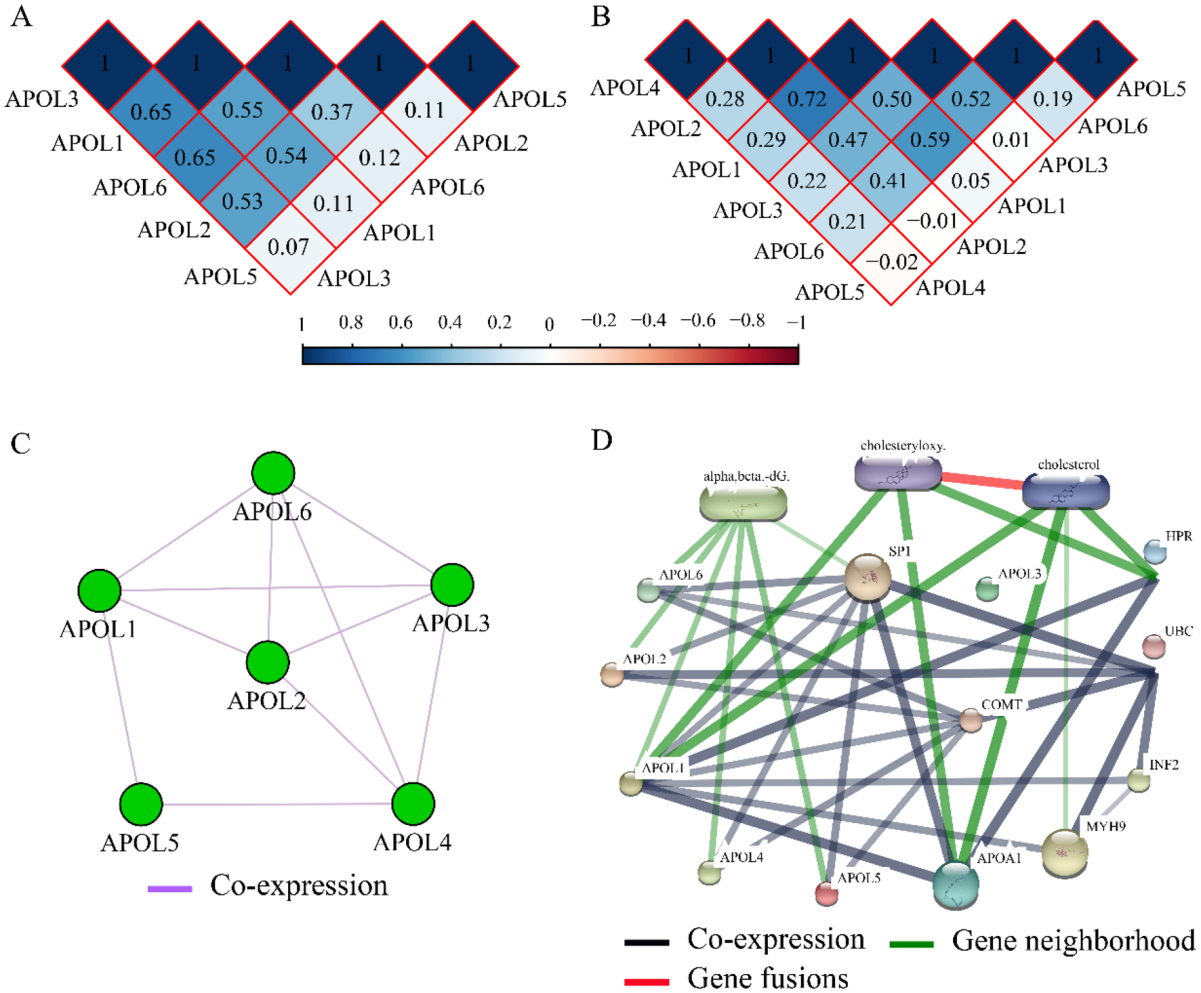
Co-expression matrix and protein-chemical compound interaction networks for APOL1-6. (**A**): Co-expression matrix for APOL1, 2, 3, 5, and 6 in the GSE14520 cohort. (**B**): Co-expression matrix for APOL1-6 in the TCGA cohort/ (**C**): Co-expression network for APOL1-6 genes. (**D**): Protein-chemical compound interaction networks for APOL1-6 and compounds.

Co-expression matrixes of APOL isoforms indicated that all five APOL isoforms were positively correlated in the GSE14520 cohort. All other isoforms were positively correlated except for a negative correlation among APOL5, APOL2, and APOL4 in TCGA cohort (Fig. 11A,B). All these genes were co-expressed at the gene level (Fig. 11C). Protein-chemical interaction networks revealed that these proteins were also co-expressed at the protein level and were associated with α, β-dG, cholesteryloxy, and cholesterol in gene neighborhoods (Fig. 11D). In addition, visualized GO terms enriched by APOLs were indicated and involved in lipoprotein binding, extracellular region, lipoprotein metabolic process, et al. (Figure S5).

### Validation of diagnostic analysis and prognosis significance by Oncomine and HCCDB databases

**Figure 12:**
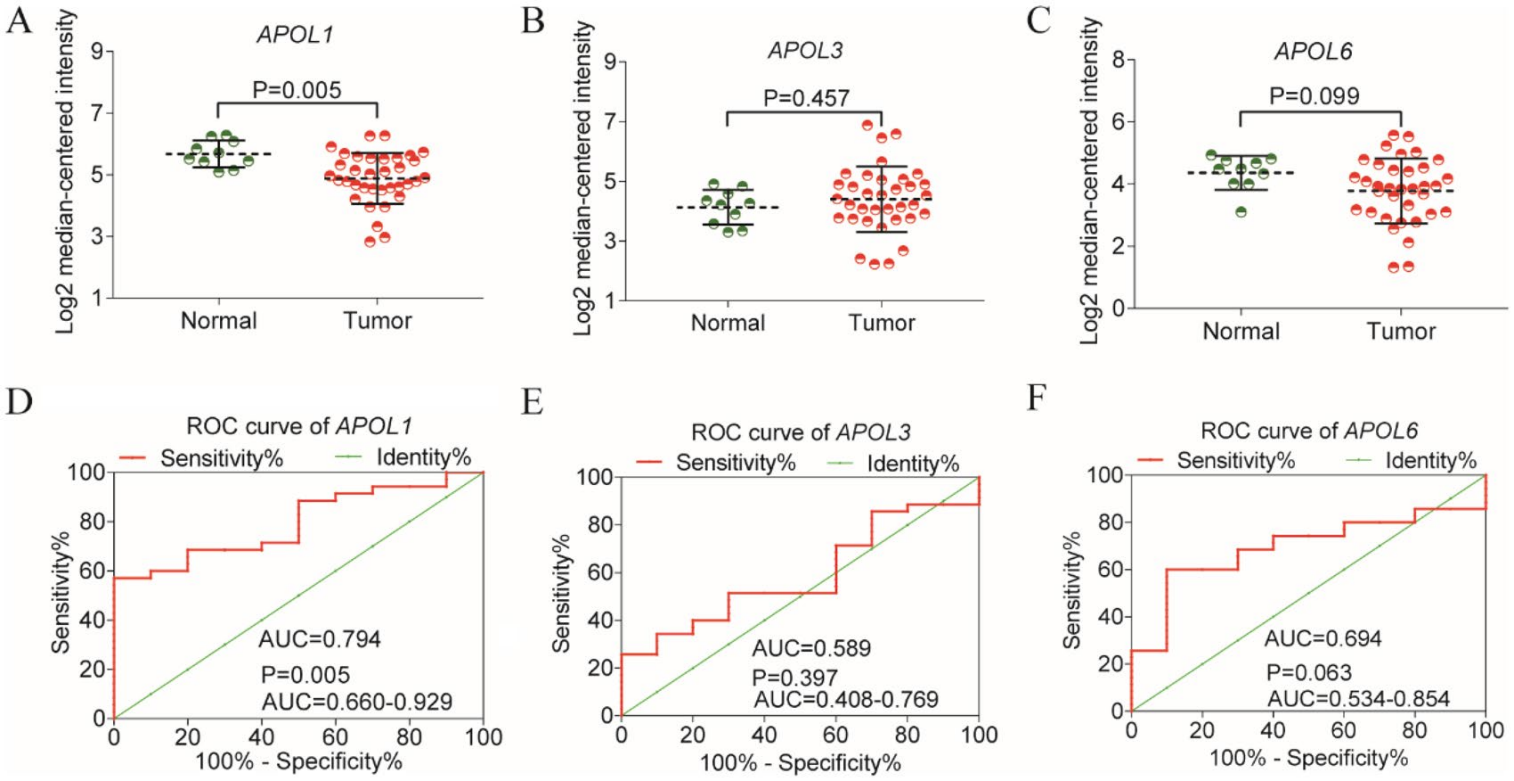
Validation of differential expressions and diagnostic values by Oncomine database for APOL1, 3 and 6. (**A**–**C**): Differential expression results in Oncomine database for APOL1, 3 and 6. (**D**–**F**) Diagnostic ROC curves for APOL1, 3 and 6 in Oncomine database.

**Figure 13:**
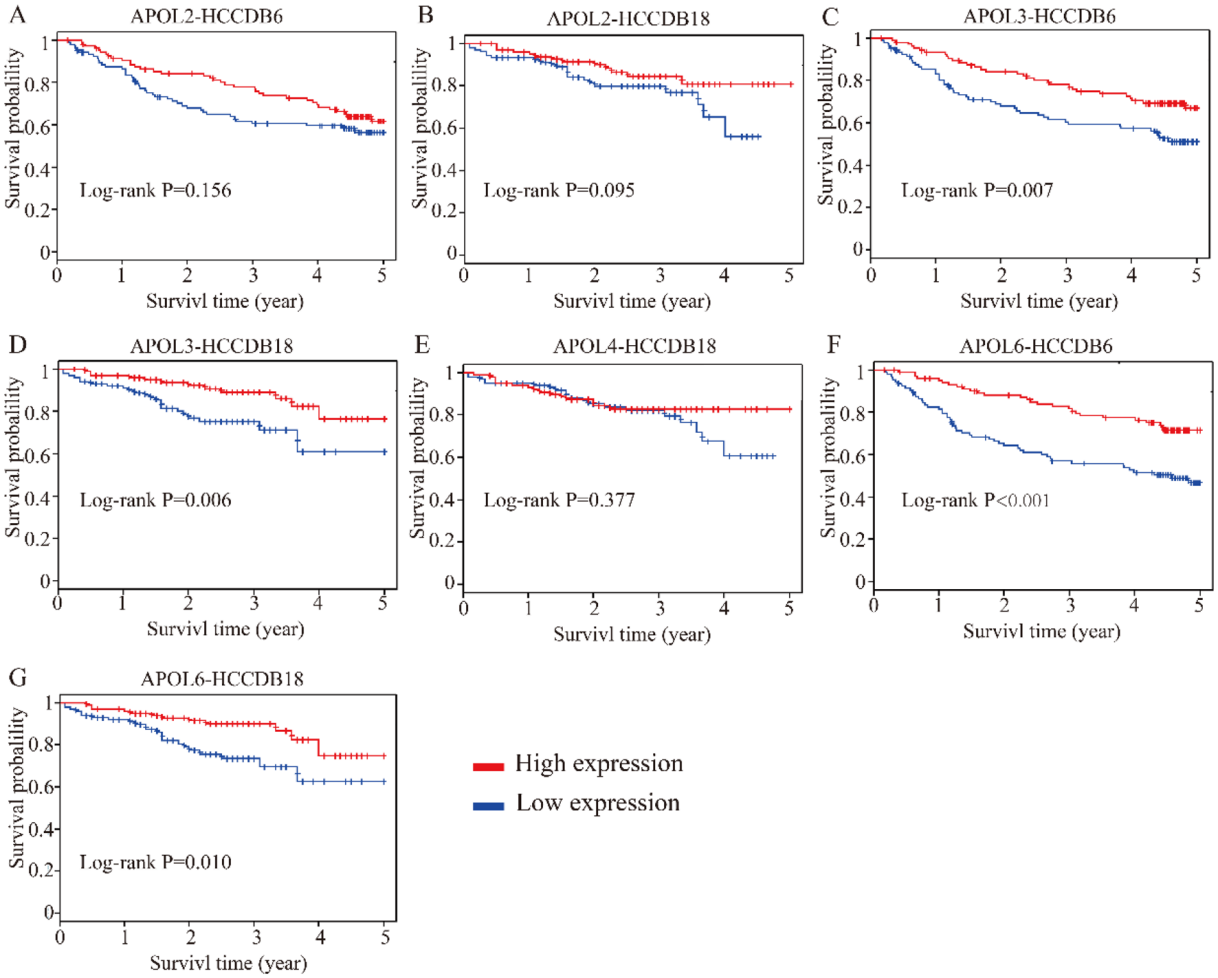
Validation of survival analysis of APOL2, 3, 4 and 6 in HCCDB database. (**A**–**B**): Survival analysis of APOL2 in HCCDB6 and HCCDB18 datasets. (**C**–**D**): Survival analysis of APOL3 in HCCDB6 and HCCDB18 datasets. (**E**): Survival analysis of APOL4 in HCCDB18 dataset. (**F**-**G**): Survival analysis of APOL6 in HCCDB6 and HCCDB18 datasets.

Differential expressions and diagnostic values of APOL1 and APOL3 were consistently validated in Oncomine database (AUC = 0.794, 0.589, Fig. 12A,C,D,F). Oncomine is a classic sample database in the field of cancer and can perform expression data, expression characteristics, gene set modules, etc. We applied it for validation of gene expression data. Strangely, APOL6 was showed weak diagnostic values in Oncomine database (AUC = 0.694, Fig. 12B,E). Furthermore, prognosis-related APOL isoforms were further validated in HCCDB database. HCCDB to serve as a one-stop online resource for exploring HCC gene expression with user-friendly interfaces, with integrating data from TCGA and GTEx. We applied it for validation of prognostic significance. APOL3 and APOL6, consistent in both TCGA and GSE14520 dataset, showed prognostic significance in two datasets of HCCDB as well (Log-rank *P* = 0.007, 0.006, Fig. 13C,D; Log-rank *P* < 0.001, = 0.010, Fig. 13F,G). However, APOL2 and APOL4, prognosis-related significance in TCGA or GSE14520 dataset, did not show prognostic significance (all *P* > 0.05, Fig. 13A,B,E).

## Discussion

We explored prospective diagnostic capacity and prognostic significance and the mechanisms of APOL isoform involvement in HCC using GSE14520 and TCGA cohorts. We found that APOL1, 3, and 6 were differentially expressed in tumor and non-tumor tissues in both cohorts. In addition, both APOL1 and APOL6 had diagnostic abilities for HCC in the cohorts. In addition, diagnostic values of APOL1 and weak APOL6 were validated in Oncomine database. APOL3 and APOL6 showed prognostic significance for OS whereas APOL2, APOL3 and APOL6 showed prognostic significance for RFS in the GSE14520 cohort. However, APOL6 showed prognostic significance for OS whereas APOL3 and APOL4 showed prognostic significance for RFS in the TCGA cohort. These results indicated that APOL6 might be associated with OS and APOL3 might be associated with RFS of HCC patients. Moreover, APOL3 and APOL6, prognosis-related significance in both TCGA and GSE14520 datasets, were consistently validated their prognostic significance in HCCDB database; whereas APOL2 and APOL4, prognosis-related significance in TCGA or GSE14520 dataset, were not validated in HCCDB database. Prospective molecular mechanism exploration suggested that APOL3 and APOL6 might participate in HCC initiation and progression via the humoral immune response, regulation of the inflammatory response, cytokine-mediated signaling pathways, chemokine signaling pathways, fatty acid metabolism, and cell adhesion molecular cams. We constructed and used risk score models and nomograms to predict the survival of HCC patients using prognosis-related genes and clinical factors. We constructed co-expression interaction networks of APOL isoforms and visualized prospective GO-term networks. RT-PCR was performed on differentially expressed genes for APOL1, 3 and 6 and to validate the diagnostic ability of APOL1 and 6.

The six APOL isoforms are a cluster spanning a region of 619 kb on chromosome 22^10^. Lowry et al. found that APOL proteins are expressed in human placenta in a study identifying novel diagnostic biomarkers for pregnancy pathologies^10^. The liver is the main source of APOL1 proteins and has highly efficient secretory activity^13^. APOL1 has been widely studied for its expression and variants with many diseases, especially with kidney diseases. Coding variants within the APOL1 gene have a high frequency in recent populations of African ancestry and the highest odds ratio association with complicated renal diseases^34, 35^. APOL1 is a trypanolytic factor that confers resistance to Trypanosoma brucei brucei, containing Trypanosoma brucei rhodensience and Trypanosoma brucei gambsience parasites^36, 37^. Trypanosoma brucei rhodensience is found mainly in Eastern and Southern Africa while Trypanosoma brucei gambsience is found mainly in Western Africa^38^. APOL1 is suggested to function in natural selection due to the unique traits of these parasites in sub-Saharan Africa^34^. APOL1 variations increase the risk of kidney diseases in African Americans. Nonsynonymous variants coded by G1 and the coding region deletion G2 in APOL1 are sequence variants that have strong relationships to focal segmental glomerulosclerosis and hypertension-attributed end-stage kidney disease. After controlling for risk variants in APOL1, the association between kidney diseases and MYH9 sequence variants^34^.

Studies conducted for the Jackson Heart Study and Women’s Health Initiative indicated that a person with two risk variants may have a twofold risk for cardiovascular disease, even though these studies had no data on mortality and contained only 12 patients with myocardial infarction^39^. A prospective investigation conducted over two decades in older adults suggested that the APOL1 genotype was associated with albuminuria, peripheral atherosclerosis, risk of myocardial infarction and death^40^. Evidence indicates that APOL1 mRNA and protein are expressed in podocytes, renal tubule cells, and glomerular endothelial cells^41, 42^. APOL1 protein is also expressed in the blood, which may be the reason for its significance in pathology^12^. However, little is known about the association between APOL1 expression or genetic variants with malignancies. Our study demonstrated that APOL1 mRNA was not associated with HCC prognosis but showed a strong diagnostic ability for HCC.

APOL2 protein expression is markedly stimulated by interferon-γ in normal human bronchial epithelial cells while APOL2 mRNA is increased in normal human lung fibroblasts and smooth muscle cells^16^. Lowering expression of APOL2 with siRNA facilitates cytotoxicity induced by interferon-γ, with a significant drop in cell viability via MTT and CyQUANT NF cell proliferation assays and an increase in hypodiploid sub-G1 cell distribution in cell cycle assays^16^. Furthermore, depletion of APOL2 promotes membrane damage, DNA fragmentation and chromatin condensation induced by interferon-γ by Hoechst and propidium iodide-double staining, DNA laddering assays and transmission electron microscopy ^16^. These findings indicate a new function for APOL2: anti-apoptotic ability in human bronchial epithelial cells from cytotoxic effects by interferon-γ and maintaining airway epithelial layer integrity^16^. Tsuang et al. found that APOL1, 2 and 4 genes are located on chromosome 22q12.3–13.1 and upregulated in brains of schizophrenic patients^18^. They conducted a family-based association study using 130 single nucleotide polymorphisms (SNPs) in APOL1-6 family members in 112 African-American, 114 European-American, 109 Chinese and 42 Japanese families with schizophrenia. They concluded that seven SNPs in APOL1, 2 and 4 are associated with schizophrenia in these families^18^. Similar to APOL1, few reports are available about APOL2 and malignancies. Our study found APOL2 mRNA was associated with HCC recurrence in an HBV-related HCC cohort. In addition, APOL4 mRNA was associated with HCC recurrence in a TCGA cohort but not in a GSE14520 cohort. Due to the inconsistence of APOL2 and APOL4 in two cohorts, we did not perform GSEA for mechanical exploration. As the above literatures mentioned, literature reports of several SNP of them, anti-apoptotic and cell proliferation ability of APOL2, and our findings would be a direction of future study concerning APOL2 and APOL4 in HCC prognosis and mechanical pathways.

APOL3 was a risk locus in a family-based association analysis of 42 hereditary prostate cancer families^17^. In addition, APOL3 is differentially expressed in tumors and controls in oral squamous cell carcinoma. This information might be helpful for selecting possible biomarkers for oral squamous cell carcinoma^43^. However, APOL3 expression in HCC has not been reported. Our study found that APOL3 was associated with HCC prognosis and recurrence. In addition, mechanism exploration suggested that APOL3 involvement in HCC might be via the humoral immune response, regulation of the inflammatory response, cytokine-mediated signaling pathways, chemokine signaling pathways, fatty acid metabolism, and cell adhesion molecular cams. The above literature indicated rs2097465 and rs132656 located within the APOL3 were associated with prostate cancer initiation as well as differential APOL3 was involved in immune response in oral squamous cell carcinoma. This suggests further concentration on rs2097465 and rs132656 loci as well as immune response of APOL3 in HCC initiation and progression.

A SNP of APOL5, rs2076672, was identified by parallel independent component analysis linked to structural components in a European-American study with 18 schizophrenia patients and 33 healthy control individuals^44^. A new locus, rs2016586 of APOL5, has a suggestive association with childhood body mass index^45^. APOLs have documented associations with HDL, rather low or very low-density lipoproteins. Wu et al. found that APOL SNPs are not associated with low-density lipoprotein cholesterol levels in a physiogenomic analysis of statin-treated^46^. To date, APOL5 has not been associated with tumors. We found that APLO5 mRNA was not associated with HCC diagnosis and prognosis.

APOL6 was identified as a novel Bcl-2 homology 3-only protein in a mining-approach using public databases^47^. Overexpression of wild-type APOL6 induces mitochondria-mediated apoptosis in p53-null colorectal cancer cells, characterized by the release of cytochrome c and Smac/DIABLO from mitochondria and activation of caspase-9^47^. Hu et al. showed that APOL6 is a downstream target of interferon-γ and upregulated by interferon-γ, which sensitizes atherosclerotic lesion-derived cells to Fas-induced apoptosis^48^. APOL6 expression partly co-localizes with activated caspase 3 in activated smooth muscle cells in atherosclerotic lesions and promotes reactive oxygen species generation, caspase activation, and apoptosis^48^. Furthermore, APOL6-induced cell apoptosis might be a potential therapeutic target for treating atherosclerosis and cardiovascular disease^48^. Apart from these studies, APLO6 has not been reported it with other tumors. Our study demonstrated that APOL6 was associated with HCC prognosis and might be a potential diagnostic biomarker for HCC. Mechanism exploration indicated involvement in HCC might be via the immune effector response, B-cell mediated immunity, cytokine-mediated signaling pathways, JAK-STAT signaling pathways, and cell adhesion molecular cams. The above studies indicated APOL6 mainly play its role via Fas-induced and mitochondria-mediated apoptosis, reactive oxygen species generation, and caspase activation. Taking present study and previous reports, further studies concerning APOL6 should be mainly focused on immune response and cell apoptosis aspects.

Apart from present study of APOLs in HCC diagnosis and prognosis, Xiaofeng Wang et al. reported HSP90α^49^, exosomal hnRNPH1^50^, circulating tumor cells^51^ and Glypican-3^52^, etc. in HCC diagnosis. Wang et al. reported cirRNA cirRHOT1^53^, cirPRKCI^54^, circulating tumor cells^51^, GALAD model^55^. Even though many attempts on HCC early diagnosis and prognostic surveillance, mostly used for early diagnosis still AFP, and (or) AFP-L3, PIVKA-II^56^ and surveillance using abdominal ultrasound every 6 months^57^.

Although we report associations between APOL isoform expression and HCC patients, our study had some limitations. First, other cohorts are needed to validate the significance of APOL isoforms with HCC patients, especially the diagnosis- and prognosis-related genes. Second, prognosis-related genes need further in vivo and in vitro functional trials, especially focusing on immune response, cell apoptosis-related pathways, caspase cascades, cytokine-medicated pathways, to explore their concrete mechanisms of involvement in HCC. Third, potential target drugs need to be explored for APOL targets for future HCC treatment of medical community. Then, both diagnostic and prognostic biomarkers should be further validated in more medical centers. Then, a combination of biomarkers with AFP (or) AFP-L3, PIVKA-II and abdominal ultrasound for early diagnosis and surveillance is novel clue for future direction.

## Conclusion

This study explored prospective diagnostic capacity and prognostic significance as well as mechanisms of APOL isoforms involvement in HCC. We found that APOL1, 3, and 6 were differentially expressed in tumor and non-tumor tissues. Both APOL1 and APOL6 had diagnostic ability for HCC in TCGA and GSE14520 cohorts. These findings were validated by Oncomine database. Prognostic significance analysis indicated that APOL6 was associated with OS and APOL3 was associated with RFS of HCC patients in both TCGA and GSE14520 datasets. And their prognostic significance was further consistently validated in HCCDB database as well. Prospective molecular mechanism exploration suggested that APOL3 and APOL6 were associated with HCC prognosis via the immune response, inflammatory response, cytokine-mediated signaling pathways, and fatty acid metabolism. We constructed and used risk score models and nomograms to predict the survival of HCC patients using prognosis-related genes and clinical factors.

### Supplementary Information

Supplementary Information.

## Data Availability

The datasets analyzed during the current study are available from the corresponding author on reasonable request.

## Abbreviations

HCC: Hepatocellular carcinoma
APOL: Apolipoprotein
HBV: Hepatitis B virus
AUC: Area under curve
TCGA: The cancer genome atlas
BCLC: Barcelona clinic liver cancer
KEGG: Kyoto encyclopedia of genes and genomes
GSEA: Gene set enrichment analysis
BP: Biological process
CC: Cellular component
MF: Molecular function
GO: Gene ontology
OS: Overall survival
MST: Median survival time
CI: Confidence interval
HR: Hazard ratio
ROC: Receiver operating characteristic
GGI: Gene-gene interaction
DAVID: Database for annotation, visualization and integrated discovery
