# Supplementary material for: Identification and validation of candidate clinical signatures of apolipoprotein L isoforms in hepatocellular carcinoma
Source: Sci Rep. 2023 Nov 28;13:20969. doi: 10.1038/s41598-023-48366-0 (PMC10684526; doi:10.1038/s41598-023-48366-0)

Supplementary Table 1. Joint-effect analysis of *APOL3* and *APOL4* for recurrence-free survival in TCGA cohort

Group	*APOL3*	*APOL4*	Recurrence-free survival			
			Events /total[∮]	MST (Days)	Adjusted HR (95%CI)	Adjusted *P* value[£]
■	Low	Low	46/93	656	Reference	**0.030**
■■	High	Low	58/135	892	0.858 (0.523-1.408)	0.545
	Low	High				
■■■	High	High	35/90	1432	**0.483 (0.279-0.839)**	**0.010**

Note: ∮ : 52 data were missing. £: P values were adjusted for HBV infection, tumor stage, vascular invasion, and radical resection; Bold indicates significant P values.

Abbreviations: NA: not available; MST: median survival time; HR: hazard ratio; 95%CI: 95% confidence interval.

Supplementary Table 2. Prognostic analysis of risk score model in GSE14520 and TCGA cohort

Cohort	Type	Group	Prognosis			
			Events /total	MST (Months)	Adjusted HR (95%CI)	Adjusted *P* value&
GSE14520	OS	Low risk	28/106	NA	Reference	**0.002**
		High risk	54/106	53.3	**2.101 (1.317-3.349)**	
GSE14520	RFS	Low risk	48/106	59.5	Reference	**0.004**
		High risk	68/106	23.6	**1.739 (1.192-2.537)**	
TCGA	RFS	Low risk	65/161	1032	Reference	**0.028**
		High risk	74/157	658	**1.609 (1.051-2.461)**	

Note: &: P values were adjusted for gender, cirrhosis and BCLC stage of OS and were adjusted for gender, cirrhosis and and BCLC stage of RFS in GSE14520 cohort and were adjusted for HBV infection, vascular invasion, tumor stage and radical resection of RFS in TCGA cohort; Bold indicates significant P values.

Abbreviations: RFS: recurrence-free survival; OS: overall survival; NA: not available; MST: median survival time; HR: hazard ratio; 95%CI: 95% confidence interval; OS: overall survival; RFS: recurrence-free survival.

Supplementary Figures

Figure S1. Joint-effect analysis for APOL2, 3, 4 and 6 for overall survival and recurrence-free survival in the GSE14520 and TCGA cohorts. A: Joint-effect analysis for APOL3 and 6 for overall survival in the GSE14520 cohort. (B-E): Joint-effect analysis for APOL2, 3 and 6 for recurrence-free survival in the GSE14520 cohort. F: Joint-effect analysis for APOL3 and 4 for recurrence-free survival in the TCGA cohort.

Figure S2. Gene ontology and KEGG pathway results for APOL6 in the GSE14520 cohort. (A-L): Gene ontology results for the APOL6 gene. (M-P): KEGG pathway results for the APOL6 gene.

Figure S3. Gene ontology and KEGG pathway results for APOL6 in the TCGA cohort. (A-L): Gene ontology results for the APOL6 gene. (M-P): KEGG pathway results for the APOL6 gene.

Figure S4. Risk score model, Kaplan-Meier plots and time-dependent receiver operative characteristic curves for overall survival in the GSE14520 cohort. A: Risk score model with risk score, survival status, and heatmap of APOL3 and 6. B: Kaplan-Meier plots by low and high overall survival-risk groups. C: Time-dependent receiver operative characteristic curves for overall survival at 1, 2, 3, 4, and 5 years.

Figure S5. Visualized gene ontology terms using APOL1-6 genes. A: Visualized biological processes using APOL1-6 genes. B: Visualized cellular components using APOL1-6 genes. C: Visualized molecular function using APOL1-6 genes.

Supplementary Figure 1.

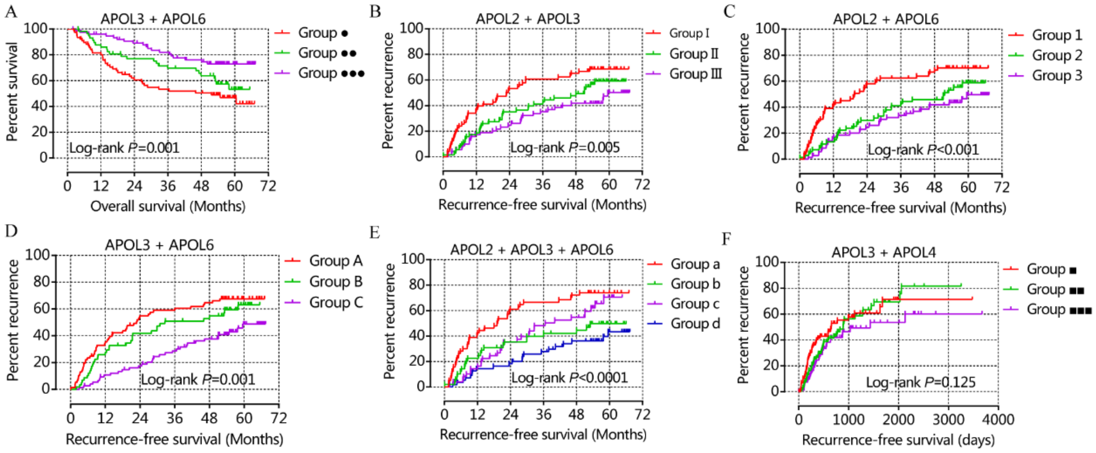

Supplementary Figure 2.

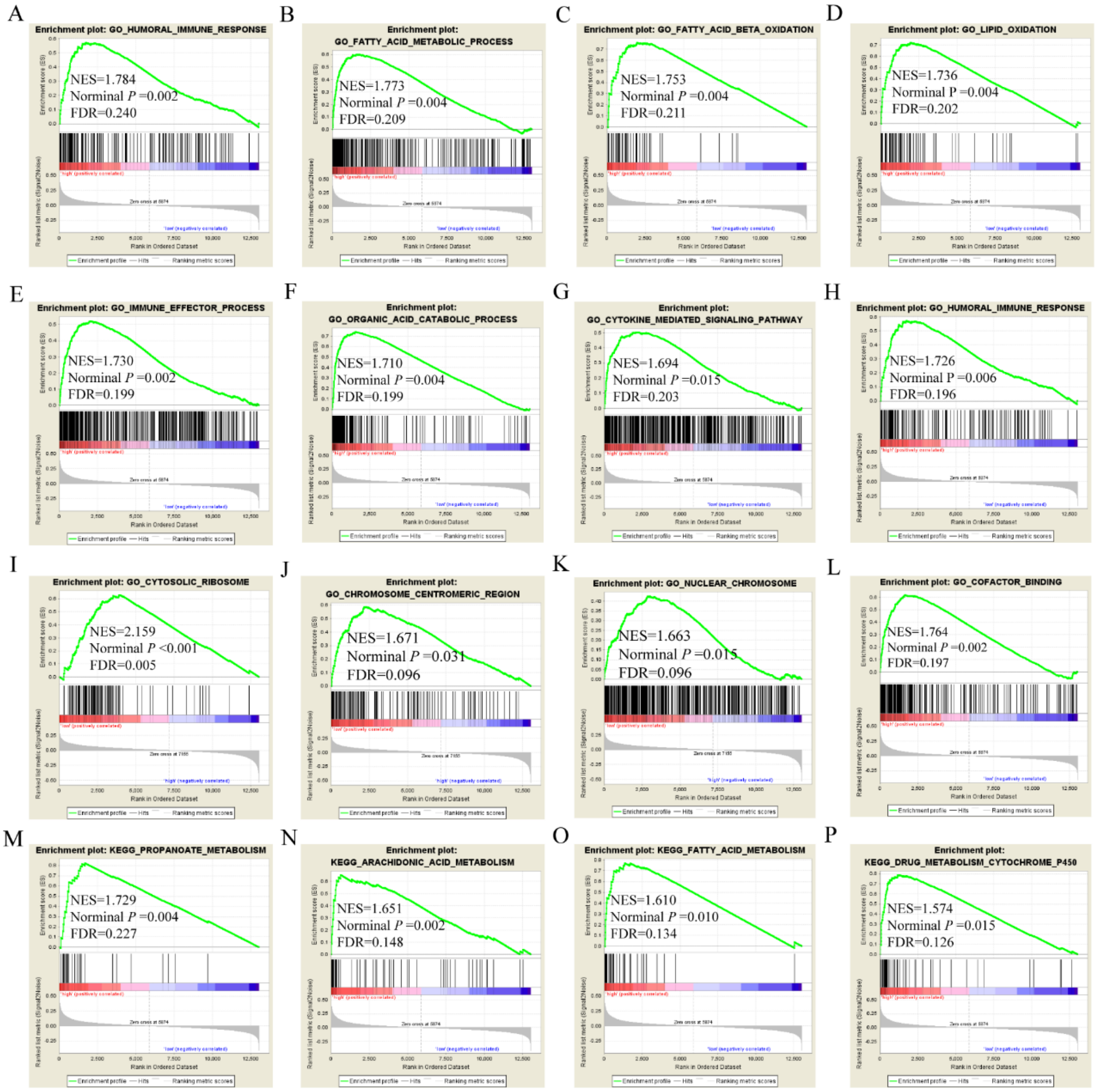

Supplementary Figure 3.

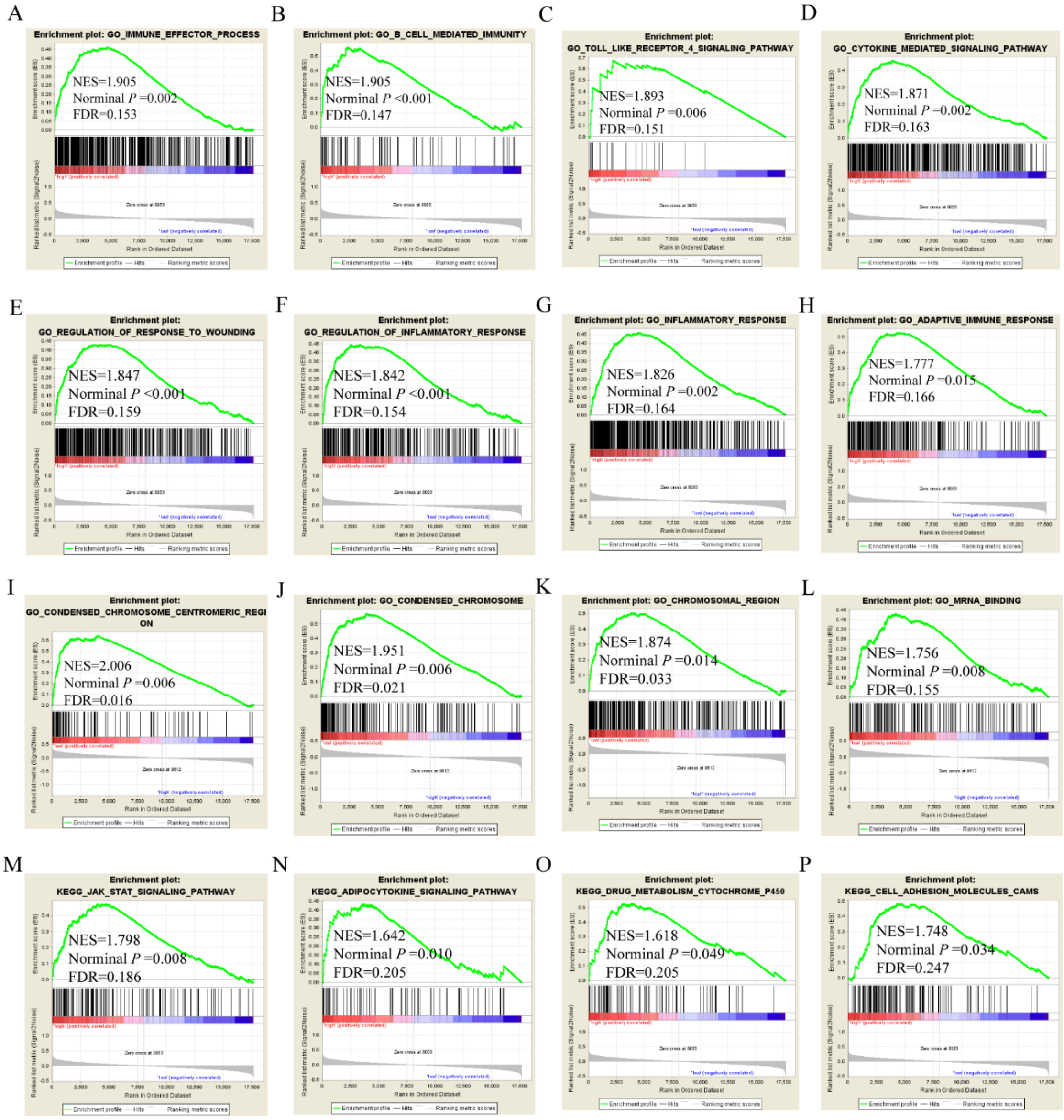

Supplementary Figure 4.

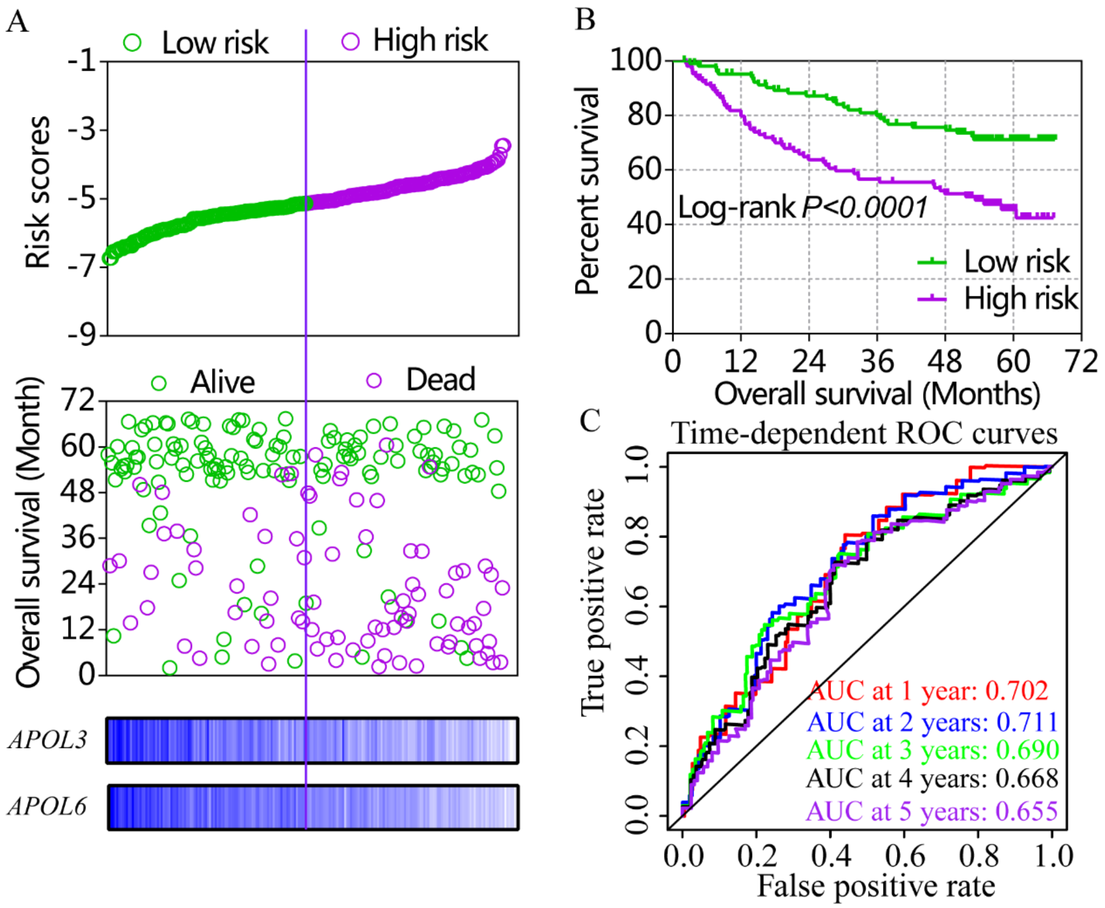

Supplementary Figure 5.

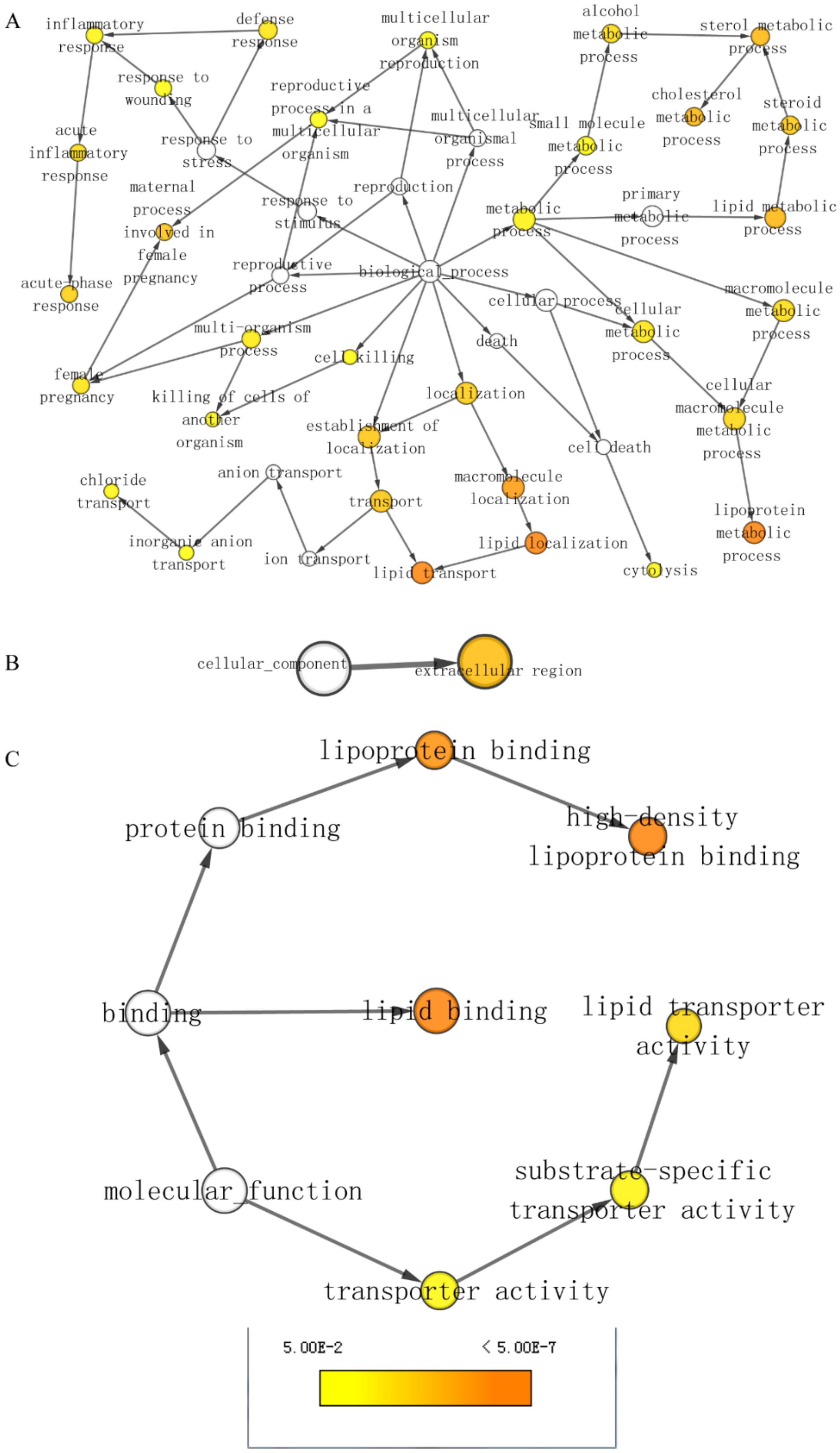

Supplement: Supplementary file 1 — Supplementary Information. [file 41598_2023_48366_MOESM1_ESM.pdf]
